# Adipocyte Piezo1 mediates obesogenic adipogenesis through the FGF1/FGFR1 signaling pathway in mice

**DOI:** 10.1038/s41467-020-16026-w

**Published:** 2020-05-08

**Authors:** ShengPeng Wang, Shuang Cao, Malika Arhatte, Dahui Li, Yue Shi, Sabrina Kurz, Jiong Hu, Lei Wang, Jingchen Shao, Ann Atzberger, Zheng Wang, Changhe Wang, Weijin Zang, Ingrid Fleming, Nina Wettschureck, Eric Honoré, Stefan Offermanns

**Affiliations:** 1 Max Planck Institute for Heart and Lung Research, Department of Pharmacology, Ludwigstr. 43, 61231 Bad Nauheim, Germany; 2 Cardiovascular Research Center, School of Basic Medical Sciences, Xi’an Jiaotong University Health Science Center, Key Laboratory of Environment and Genes Related to Diseases, No.76 West Yanta Road, Yanta District, Xi’an, China; 3 Université Côte d’Azur, Centre National de la Recherche Scientifique, Institut de Pharmacologie Moléculaire et Cellulaire, Labex ICST, Valbonne, France; 4 The State Key Laboratory of Pharmaceutical Biotechnology, Department of Pharmacology and Pharmacy, The University of Hong Kong, Hong Kong SAR, China; 5 Institute for Vascular Signalling, Centre for Molecular Medicine, Goethe University, Frankfurt am Main, Germany; 6 Max Planck Institute for Heart and Lung Research, Flow Cytometry Service Group, Ludwigstr. 43, 61231 Bad Nauheim, Germany; 7 Department of Hepatobiliary Surgery, First Affiliated Hospital of Xi’an Jiaotong University, Xi’an, China; 8 Center for Mitochondrial Biology and Medicine, School of Life Science and Technology, Xi’an Jiaotong University, Xi’an, China; 9 Department of Pharmacology, School of Basic Medical Sciences, Xi’an Jiaotong University Health Science Center, Xi’an, China; 10 Center for Molecular Medicine, Goethe University Frankfurt, Theodor-Stern-Kai 7, 60590 Frankfurt, Germany

**Keywords:** Fat metabolism, Metabolic disorders

## Abstract

White adipose tissue (WAT) expansion in obesity occurs through enlargement of preexisting adipocytes (hypertrophy) and through formation of new adipocytes (adipogenesis). Adipogenesis results in WAT hyperplasia, smaller adipocytes and a metabolically more favourable form of obesity. How obesogenic WAT hyperplasia is induced remains, however, poorly understood. Here, we show that the mechanosensitive cationic channel Piezo1 mediates diet-induced adipogenesis. Mice lacking Piezo1 in mature adipocytes demonstrated defective differentiation of preadipocyte into mature adipocytes when fed a high fat diet (HFD) resulting in larger adipocytes, increased WAT inflammation and reduced insulin sensitivity. Opening of Piezo1 in mature adipocytes causes the release of the adipogenic fibroblast growth factor 1 (FGF1), which induces adipocyte precursor differentiation through activation of the FGF-receptor-1. These data identify a central feed-back mechanism by which mature adipocytes control adipogenesis during the development of obesity and suggest Piezo1-mediated adipocyte mechano-signalling as a mechanism to modulate obesity and its metabolic consequences.

## Abstract (web summary)

Adipose tissue expansion occurs via enlargement of adipocytes as well as the generation of new fat cells, the latter being associated with more favorable metabolic outcomes. Here, the authors show that activation of adipocyte Piezo1 results in release of FGF1 and stimulates the differentiation of adipocyte precursor cells.

## Introduction

The white adipose tissue (WAT) is the primary site for fat storage and release. A dysbalance of this process leads to obesity and associated diseases including insulin resistance and type-2 diabetes, which affect a growing number of people worldwide^1–4^. Despite the essential role of WAT expansion in metabolic disorders, the mechanisms of WAT growth in obesity are still incompletely understood. Under increased caloric intake, WAT expands through an increase in the size of preexisting adipocytes (hypertrophy) as well as through the formation of new adipocytes from adipocyte precursor cells (adipogenesis) resulting in an increased number of adipocytes (hyperplasia)^5–10^. The balance of adipocyte hypertrophy and hyperplasia during obesity has a strong effect on the complications of obesity including type-2 diabetes. Whereas hypertrophy promotes adipose tissue inflammation and insulin resistance, hyperplasia results in smaller adipocytes and goes along with less adipose tissue inflammation and better insulin sensitivity^7,11^.

Adipogenesis under a hypercaloric diet is rapidly initiated by increased proliferation of adipocyte precursor cells, which are immature mesenchymal cells located in the stromal, vascularized tissue between adipocytes^12–15^. The subsequent differentiation of adipocyte precursor cells into mature adipocytes takes several weeks^6,15^. Multiple signaling pathways and mediators have been described which regulate pre-adipocyte proliferation and differentiation into mature adipocytes such as bone morphogenetic protein signaling and WNT signaling or insulin-like growth factor 1^8,16–18^. It is, however, incompletely understood through which mechanisms adipocyte progenitor cell proliferation and differentiation is controlled by systemic and local factors. It is also not clear, to what degree mature adipocytes sense a metabolic overload and functionally interact with progenitor cells to regulate adipogenesis.

Piezo1 and Piezo2 are homotrimeric mechanically activated non-selective cationic channels, which mediate mechanically induced currents^19–21^. Piezo1 has been shown to be gated directly by changes in membrane tension^22–24^ and to be involved in diverse processes including endothelial flow sensing^25^, osmotic homeostasis in erythrocytes^26^ or epithelial cell density regulation^27^. Since we observed high expression of Piezo1 in mature white adipocytes we speculated that Piezo1-mediated mechanosignalling is involved in adipose tissue function and remodeling.

In this paper, we report that Piezo1 expressed in mature adipocytes is activated under a hypercaloric diet and induces adipocyte precursor differentiation through the release of the adipogenic mediator FGF1.

## Results

### Functional expression of Piezo1 in adipocytes

**Fig. 1:**
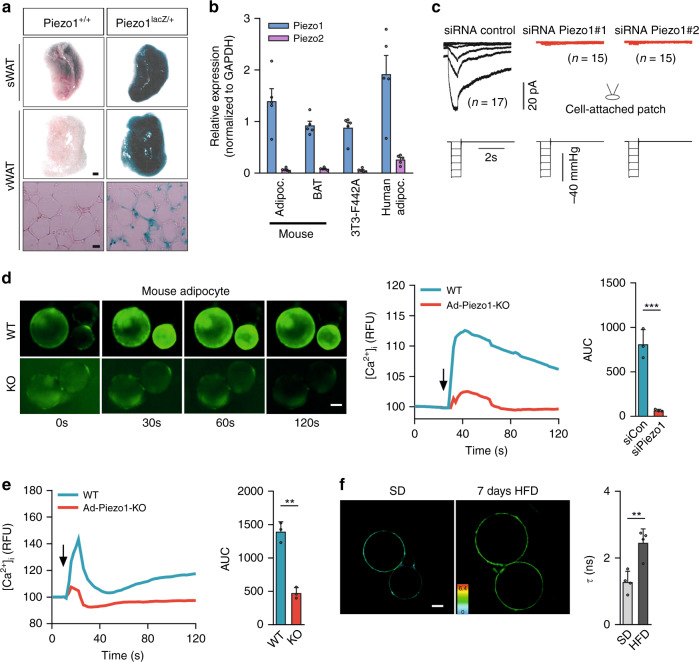
Functional expression Piezo1 in adipocytes. **a** X-gal staining of inguinal sWAT and epididymal vWAT or of histological sections of vWAT prepared from wild-type mice (Piezo^+/+^) or from Piezo1^lac*Z*/+^ mice, bar lengths: 1 mm (whole mount) and 20 μm (histological section). Shown is a representative of three independent experiments. **b** Expression of mRNAs encoding Piezo1 or Piezo2 in murine and human vWAT adipocytes, brown adipose tissue (BAT) and in differentiated 3T3-F442A adipocytes (*n* = 5 samples for each group). (**c**) Stretch-activated currents from differentiated 3T3-F442A were recorded in the cell-attached patch clamp configuration. The holding potential was −80 mV and the membrane was stretched by pulses of negative pressure with a 10 mm Hg increment applied every 10 ss. Cells were transfected with control siRNAs or with siRNAs against Piezo1. **d**, **e** Fluo-4-loaded adipocytes prepared from vWAT of wild-type (WT) or Ad-Piezo1-KO mice were exposed to 10 µM Yoda1 (**d**) or hypotonic buffer (210 mOsm/kg; **e**), and [Ca^2+^]_i_ was determined as fluorescence intensity (RFU, relative fluorescence units); shown are representative images and experiments (*n* = 3 mice per group (12 and 6 measurements per animal in **d**, **e**, respectively)). Arrows indicate the addition of Yoda1 or of hypotonic buffer. Bar diagrams show the area under the curve (AUC) of the Ca^2+^-transient. **f** Fluorescence lifetime τ_1_ images of FliptR in visceral adipocytes from wild-type mice kept under normal show or fed a HFD for 7 days. Corresponding lifetime mean values are shown in the bar diagram. The color bar corresponds to lifetime in nanoseconds (ns). *n* = 4 mice per group (10 measurements per animal) (**f**). Bar lengths in **d**, **f**: 10 μm. Shown are mean values ± s.e.m.; **P ≤ 0.01; ***P ≤ 0.001 (two-tailed non-parametric Mann–Whitney *U*-test). Source data are provided as a Source data file.

In a mouse line expressing β-galactosidase under the control of the Piezo1 promoter, we observed high expression in inguinal (subcutaneous) and epididymal (visceral) WAT (Fig. 1a). Quantitative RT-PCR and immunoblotting showed expression of Piezo1, but not of Piezo2 in mature mouse and human adipocytes from visceral WAT (vWAT) and in differentiated 3T3-F442A adipocytes (Fig. 1b and Supplementary Fig. 1a). Cell attached patch-clamp recordings of differentiated 3T3-F442A cells revealed the existence of large stretch-activated currents (Fig. 1c) with a reversal potential close to 0 mV, as expected for a non-selective cationic current, and with inactivation and deactivation kinetics remarkably slow compared to Piezo1 expressed in transfected HEK cells (Supplementary Fig. 1b, c). The knock-down of Piezo1 expression using validated siRNAs^28^ suppressed this stretch-activated current (Fig. 1c). Moreover, exposure of differentiated 3T3-F442A cells to the Piezo1 activator Yoda1 induced a transient increase in the free cytosolic Ca^2+^-concentration, which was not seen after siRNA-mediated suppression of Piezo1 expression (Supplementary Fig. 1d). Similarly, in murine adipocytes, Yoda1 and also short term exposure to a hypotonic solution induced a significant increase in the free cytosolic Ca^2+^-concentration (Fig. 1d, e), an effect which was greatly reduced in adipocytes of tamoxifen-induced adipocyte-specific Piezo1-deficient mice (Ad-Piezo1-KO) (Fig. 1d, e). Since adipocytes show a significant increase in size already after 2 days of HFD feeding (Supplementary Fig. 1e, f), we used the fluorescent lipid tension sensor, FliptR^29^ to determine membrane tension in mature adipocytes. We observed that adipocyte membrane tension significantly increases after 7 days of HFD feeding (Fig. 1f).

### Loss of adipocyte Piezo1 promotes adipocyte hypertrophy under HFD

**Fig. 2:**
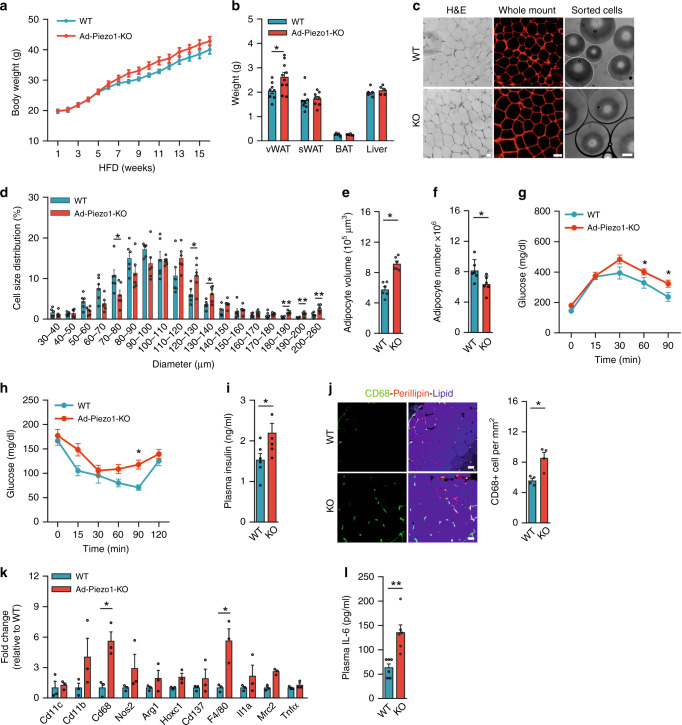
Metabolic phenotype of Ad-Piezo1-KO mice under HFD. **a** Body weight of wild-type (WT) or Ad-Piezo1-KO mice during HFD feeding for 16 weeks (*n* = 11 mice (WT), *n* = 12 mice (KO)). **b** Weight of epididymal vWAT, inguinal sWAT, BAT and the liver from wild-type (WT) or Ad-Piezo1-KO mice fed a HFD for 16 weeks (*n* = 8 mice (vWAT WT and sWAT); *n* = 5 mice (BAT WT and liver); *n* = 10 mice (vWAT KO); *n* = 6 mice (BAT KO)). **c** H&E-stained vWAT sections, whole-mount vWAT stained with anti-perilipin antibodies and sorted vWAT adipocytes from wild-type (WT) and Ad-Piezo1-KO (KO) mice; bar length: 50 μm. Shown is a representative of 4 independent experiments. **d** Distribution of size of vWAT adipocytes prepared from wild-type (WT) and Ad-Piezo1-KO mice fed HFD for 16 weeks (*n* = 6 mice (WT and KO each)). **e**, **f** Average volume (**e**) and total number (**f**) of adipocytes in epididymal vWAT from wild-type (WT) and Ad-Piezo1-KO (KO) mice (*n* = 6 mice (WT and KO each)). **g**–**i** Glucose tolerance (**g**), insulin tolerance (**h**) and plasma insulin levels (**i**) in wild-type (WT) and Ad-Piezo1-KO mice (KO) fed a HFD for 16 weeks (*n* = 6 mice (WT in **g**, WT and KO in **i**, **h**), *n* = 7 mice (KO in **g**)). **j** Whole mount of vWAT from wild-type (WT) or Ad-Piezo1-KO mice (KO) stained with anti-CD68 and anti-perilipin antibodies as well as with LipidTOX (lipid); bar length: 50 μm. The bar diagram shows the statistical evaluation of CD68-positive cells per area (*n* = 4 mice (at least 10 sections per mouse)). **k**, **l** Expression of inflammation marker genes in vWAT (**k**) and IL-6 levels in plasma (**l**) from wild-type (WT) and Ad-Piezo1-KO mice (KO) fed HFD for 16 weeks (*n* = 3 mice (WT and KO each in **k**), *n* = 6 mice (WT and KO each in **l**)). Shown are mean values ± s.e.m.; *P ≤ 0.05; **P ≤ 0.01; n.s., not significant (two-tailed non-parametric Mann–Whitney *U*-test). Source data are provided as a Source data file.

In Ad-Piezo1-KO mice, Piezo1 mRNA and protein were selectively suppressed in WAT upon induction of Cre by tamoxifen (Supplementary Fig. 1g, h) without eliciting changes in body weight, glucose tolerance and the size and number of adipocytes in the subcutaneous WAT (sWAT) and vWAT when on a standard diet (SD) (Supplementary Fig. 1i–l). When fed a HFD for 16 weeks, we observed a similar gain in body weight in control and Ad-Piezo1-KO littermates (Fig. 2a). Also the mass of sWAT, brown adipose tissue, and liver as well as the food intake, heat production, and respiratory quotient were not different between the two strains (Fig. 2b; Supplementary Fig. 2a–d). There was, however, a significant increase in vWAT mass of Ad-Piezo1-KO animals (Fig. 2b). Histological and flow cytometric analysis of the vWAT revealed an increased adipocyte sectional area and a pronounced increase in the number of large adipocytes (diameter > 110 μm) in Ad-Piezo1-KO animals (Fig. 2c–e and Supplementary Fig. 5a), whereas the total number of adipocytes in the vWAT was reduced (Fig. 2f). As described before^6,15^, HFD did not induce adipocyte hyperplasia in the sWAT (Supplementary Fig. 2e). A relative increase in adipocyte hypertrophy has been reported to result in increased insulin resistance and inflammation in humans and mice^7,30–32^. Consistent with this, Ad-Piezo1-KO mice that received HFD exhibited a significantly reduced glucose tolerance (Fig. 2g), a modest increase in insulin resistance (Fig. 2h) and increased insulin plasma levels (Fig. 2i). The same animals had an increased numbers of CD68^+^ macrophages and higher levels of inflammatory marker genes in their vWAT (Fig. 2j, k) as well as increased IL-6 plasma levels (Fig. 2l).

### Adipocyte Piezo1 is required for obesogenic adipogenesis

**Fig. 3:**
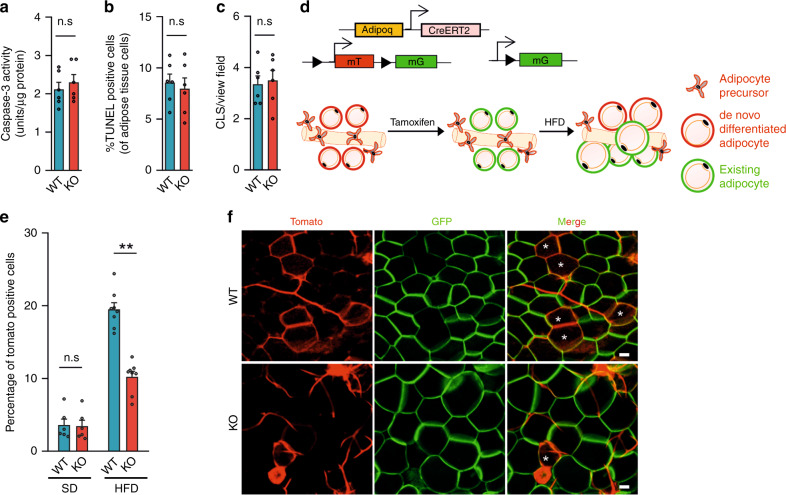
Analysis of in vivo adipogenesis in Ad-Piezo1-KO mice. **a**–**c** Caspase-3 activity (**a**), TUNEL staining (**b**) and crown-like structures (CLS; **c**) in vWAT from wild-type (WT) and Ad-Piezo1-KO mice (KO) fed HFD for 16 weeks (*n* = 6 mice (at least 10 sections per mouse in **b**, **c**)). **d** Experimental design of analysis of adipogenesis in vivo using Adipoq-CreERT2;mT/mG mice. **e**, **f** Statistical analysis (at least 20 sections per mouse; **e**) and representative images (**f**) of adipocyte tracing in Adipoq-CreERT2;mT/mG;Piezo1^flox/flox^ (KO) and Adipoq-CreERT2;mT/mG;Piezo1^+/+^ mice (WT) after tamoxifen treatment and 8 weeks of HFD or SD in vWAT (*n* = 6 mice (SD), *n* = 8 mice (HFD)); bar length: 50 μm. Shown are mean values ± s.e.m.; **P ≤ 0.01; n.s., not significant (two-tailed non-parametric Mann–Whitney *U*-test). Source data are provided as a Source data file.

The reduced adipocyte number and increased adipocyte size in vWAT from HFD-fed Ad-Piezo1-KO mice suggested either an increase in adipocyte death or a decrease in HFD-induced adipogenesis, or a combination of both. Analysis of vWAT from Ad-Piezo1-KO mice revealed no sign of increased programmed cell death compared to wild-type animals such as increased caspase-3 activity, DNA fragmentation or crown-like structures (Fig. 3a–c and Supplementary Fig. 2f). Therefore, we focused on adipocyte hyperplasia in response to HFD feeding and performed adipocyte pulse-chase experiments^15^ in adipocyte-specific, tamoxifen-inducible Cre transgenic mice (Adipoq-CreERT2) crossed with the mT/mG Cre reporter line, which switches from membrane-targeted Tomato expression to membrane-targeted GFP expression upon Cre-mediated recombination^15^ (Fig. 3d). Eight weeks after induction of Cre activity, 4% of the adipocytes in vWAT from wild-type and Ad-Piezo1-KO mice fed a SD were mTomato-positive (Fig. 3e and Supplementary Fig. 2g). In contrast, after 8 weeks of HFD vWAT from wild-type mice contained ~20% mTomato-positive adipocytes, indicating that they formed from mTomato-positive adipocyte precursor cells after tamoxifen treatment (Fig. 3e, f). However, only half of the number of mTomato-positive cells were found in Ad-Piezo1-KO mice (Fig. 3e, f). Thus, the recruitment of new adipocytes from adipocyte precursors was compromised in HFD-fed Ad-Piezo1-KO animals.

**Fig. 4:**
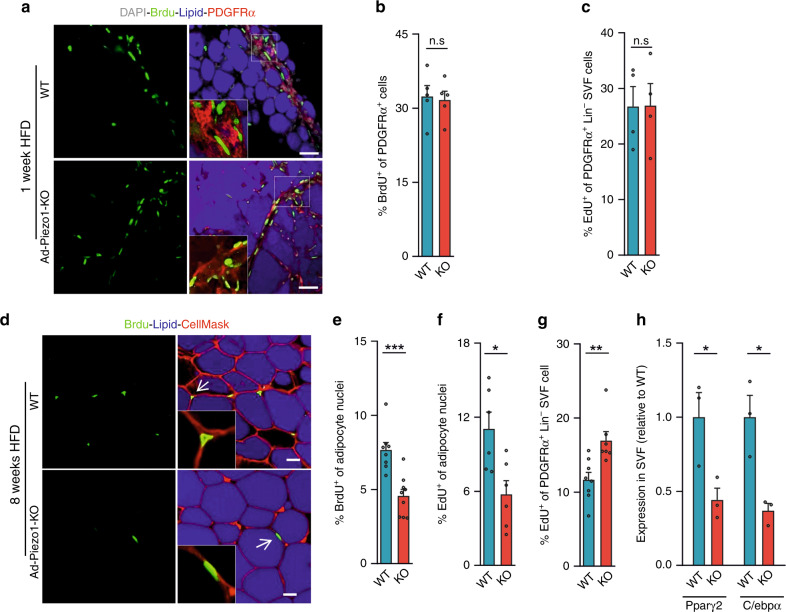
Analysis of adipocyte precursor proliferation and differentiation in Ad-Piezo1-KO mice. **a**–**g** Wild-type (WT) and Ad-Piezo1-KO mice (KO) received BrdU (**a**, **b**, **d**, **e**) or EdU (**c**, **f**, **g**) for 1 week while feeding HFD for 1 week (**a**–**c**) or 8 weeks (**d**–**g**) started. Shown are representative images (**a**, **d**) and quantifications (**b**, **e**) of immunofluorescence staining for BrdU in nuclei of SVF stained with an anti-PDGFRα antibody and LipidTOX (lipid) (**a**, **b**) or in adipocyte nuclei of vWAT (**d**, **e**). Tissues were stained with LipidTOX (lipid) and CellMask to visualize adipocyte plasma membranes. Insets show magnifications of boxed areas (**d**) or of typical adipocyte nuclei indicated by arrows (**d**); bar length: 50 μm (**a**) and 20 μm (**d**) (*n* = 5 mice (at least 12 sections per mouse in **b**), *n* = 8 or 9 mice (WT or KO in **e**; at least 15 sections per mouse)). **c**, **f**, **g** Flow-cytometric analysis of EdU incorporation in PDGFRα-positive, CD31-negative and CD45-negative (Lin^−^;PDGFRα^+^) cells of the vWAT SVF (**c**, **g**) or in adipocyte nuclei (**f**) of wild-type (WT) and Ad-Piezo1-KO mice (KO) (*n* = 4 mice (WT and KO in **c**), *n* = 6 mice (WT and KO in **f)**, *n* = 8 mice (WT in **g**), *n* = 7 mice (KO in **g**)). In **c**, **f**, **g** at least 5000 cells were analyzed per animal. **h** Expression of mRNA encoding Pparγ2 and C/ebpα in the SVF of vWAT prepared from wild-type (WT) and Ad-Piezo-KO mice (KO) fed a HFD for 16 weeks (*n* = 3 mice (WT and KO in **h**). Shown are mean values ± s.e.m.; *P ≤ 0.05; **P ≤ 0.01; ***P ≤ 0.001; n.s., not significant (two-tailed non-parametric Mann–Whitney *U*-test). Source data are provided as a Source data file.

To test whether the defect in HFD-induced adipogenesis was due to reduced adipocyte precursor proliferation or differentiation, we labeled adipocyte precursors in WAT with bromodeoxyuridine (BrdU) or ethynyl-deoxyuridine (EdU) in vivo. After 1 week of HFD feeding, when increased adipocyte precursor proliferation was already observed^15^, the number of BrdU-labeled PDGFRα-positive vWAT stromal cells did not differ between wild-type and Ad-Piezo1-KO mice (Fig. 4a, b). Also, flow cytometric analysis of EdU incorporation into highly enriched Cd31^−^;Cd45^−^;PDGFRα^+^ (Lin^−^;PDGFRα^+^) adipocyte precursor cells showed no difference (Fig. 4c and Supplementary Fig. 5b). In contrast, after 8 weeks of HFD feeding, when BrdU-labeled adipocyte precursors differentiate into mature adipocytes^15^, the number of BrdU-positive adipocytes in vWAT of Ad-Piezo1-KO mice was decreased by ~50% (Fig. 4d, e), a finding confirmed by flow cytometric analysis of EdU incorporation into adipocyte nuclei (Fig. 4f and Supplementary Fig. 5c). Numbers of EdU-positive Lin^−^;PDGFRα^+^ precursor cells in the SVF on the other hand were increased in Ad-Piezo1-KO mice (Fig. 4g and Supplementary Fig. 5b). We also found decreased expression of the early adipocyte differentiation markers Pparγ2 and C/ebpα in the SVF of HFD-fed Ad-Piezo1-KO mice (Fig. 4h). Thus, the loss of Piezo1 in mature adipocytes has no effect on adipocyte precursor proliferation, but strongly affected their differentiation into new adipocytes.

### Piezo1 induces adipocyte precursor differentiation via FGF1

**Fig. 5:**
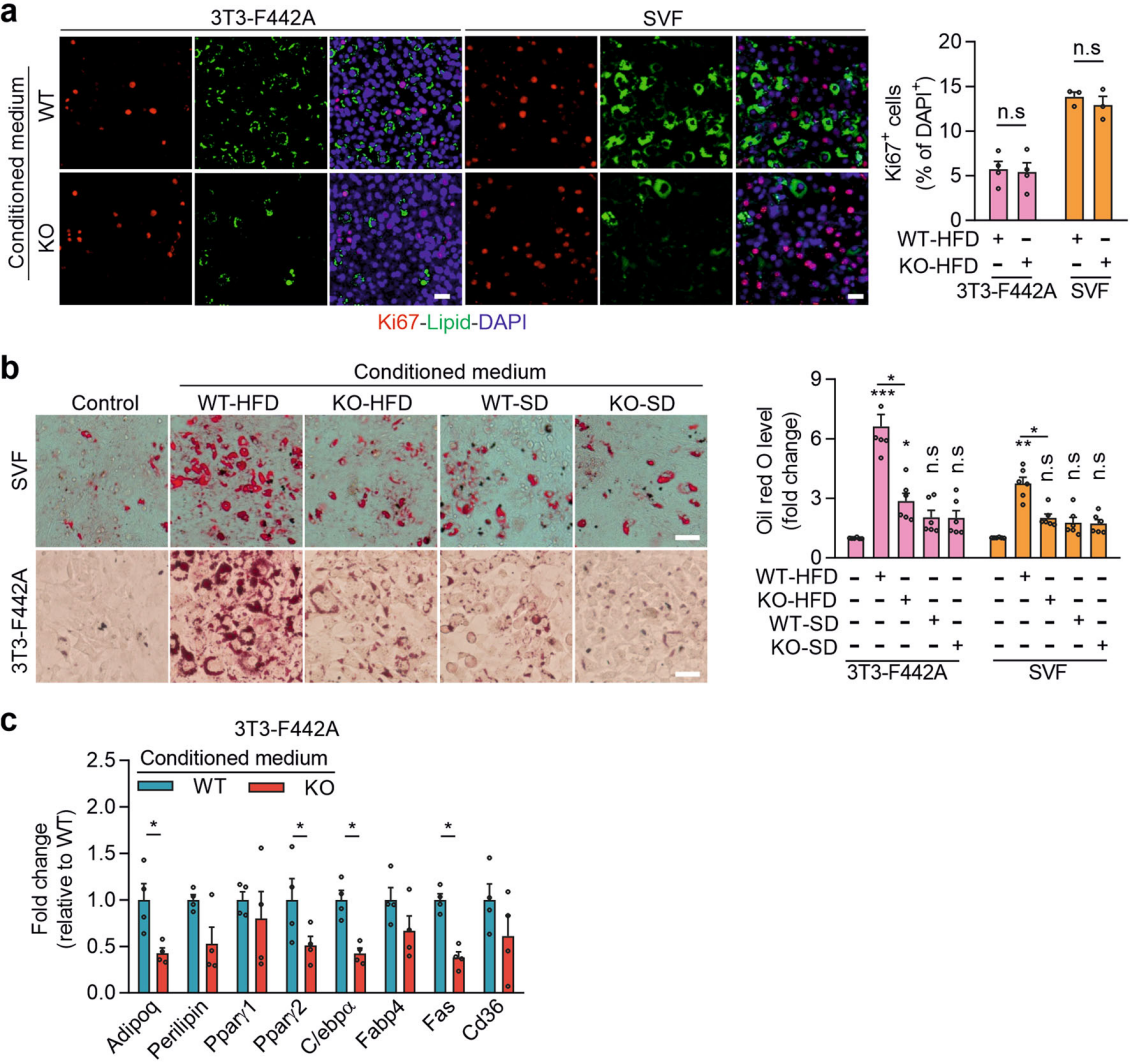
Effect of conditioned medium from vWAT adipocytes on preadipocyte proliferation and differentiation. **a**–**c** Effects of conditioned medium from vWAT adipocytes prepared from wild-type (WT) and Ad-Piezo1-KO (KO) mice fed a HFD (**a**–**c**) or SD (b) for 16 weeks or of unconditioned medium (control in **b**). In **a**, proliferation of 3T3-F442A cells or of cells of the SVF was visualized by staining for Ki67, and cells were counterstained with DAPI and BODIPY (lipid). Shown are representative images and the statistical evaluation (*n* = 4 mice (WT and KO, 3T3-F442A), *n* = 3 mice (WT and KO, SVF); at least 10 fields were analyzed per mouse). Bar length: 50 μm. In **b**, SVF cells or undifferentiated 3T3-F442A cells were exposed to the medium and stained with Oil-red-O. Shown are representative images and the statistical evaluation of Oil-red-O content (*n* = 6 mice (for all groups)); bar length: 50 μm. In **c**, expression of different adipocyte marker genes in 3T3-F442A cells (*n* = 4 mice (for all groups)) was determined. Shown are mean values ± s.e.m.; *P ≤ 0.05; **P ≤ 0.01; ***P ≤ 0.001; n.s., not significant (compared to control in **b**) (two-tailed non-parametric Mann–Whitney *U*-test). Source data are provided as a Source data file.

**Fig. 6:**
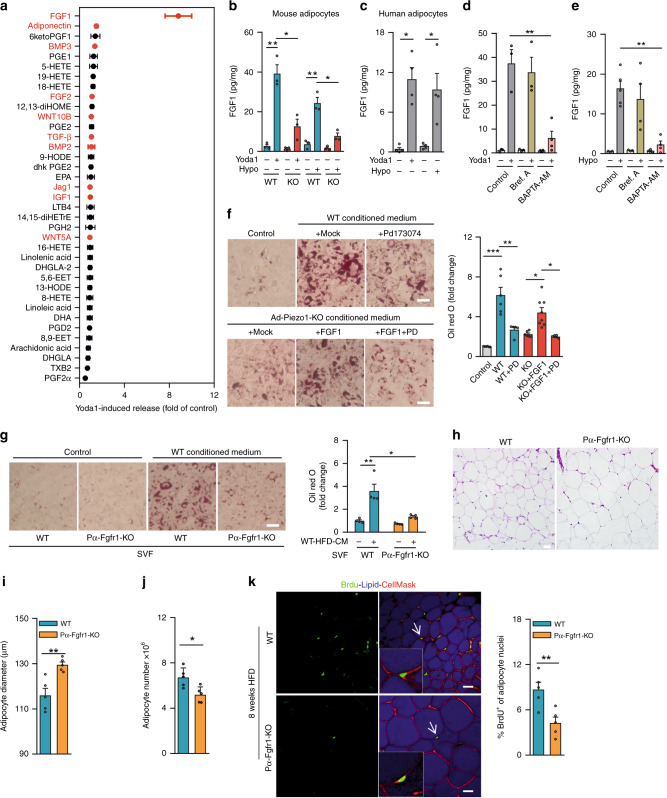
FGF1 mediates Piezo1-dependent regulation of adipocyte differentiation. **a** Yoda1 (10 μM) effect on release of protein mediators (red) and lipids (black) from mouse adipocytes (ratio of release in the presence and absence of Yoda-1 (*n* = 5 mice)). **b**, **c** Effect of Yoda1 (10 µM) or hypotonic buffer (hypo; 210 mOsm/kg) on FGF1 release from vWAT adipocytes from wild-type (WT) and Ad-Piezo1-KO mice (KO) (**b**) or from human vWAT (**c**) (*n* = 3 mice (**b**), *n* = 4 samples (**c**)). **d**, **e** Effect of Yoda1 (10 μM; **d**) or hypotonic buffer (hypo; 210 mOsm/kg; **e**) on FGF1 release from mouse adipocytes pretreated in the absence or presence of brefeldin A (bref. A) or BAPTA-AM (*n* = 3 mice (control and bref. A in **d** as well as control and control + bref. A in **e**); *n* = 5 (control + hypo in **e**); *n* = 4 mice (BAPTA in **d** and all other groups)). **f** PD173074 (PD; 10 μM) effect on 3T3-F442A cell differentiation induced by conditioned medium of vWAT adipocytes from wild-type mice fed HFD for 16 weeks and effect of FGF1 (10 ng/ml) and FGF1 + PD173074 on differentiation of 3T3-F442A cells exposed to conditioned medium from vWAT adipocytes from KO animals. Control: non-conditioned medium. Cells were stained with Oil-red-O. **g** Differentiation of SVF cells from wild-type or Pdgfrα-CreERT2;Fgfr1^flox/flox^ (Pα-Fgfr1-KO) mice exposed to conditioned medium from wild-type adipocytes from HFD-fed mice (Oil-red-O staining; *n* = 4 mice per condition). **h** H&E-stained vWAT sections from wild-type (WT) and Pα-Fgfr1-KO mice fed a HFD for 12 weeks (one representative of three independent experiments). **i**, **j** Average diameter (**i**) and total number (**j**) of adipocytes in vWAT from wild-type (WT) and Pα-Fgfr1-KO mice fed a HFD (*n* = 5 mice each). **k** Wild-type (WT) and Pα-Fgfr1-KO mice received BrdU during the first week of an 8-week HFD feeding period. Representative images and quantifications of immunofluorescence staining for BrdU in adipocyte nuclei of vWAT after HFD feeding (*n* = 5 mice (≥15 sections per mouse)). Bar lengths in **f**, **g**, **h**, **k**: 50 μm. Shown are mean values ± s.e.m.; *P ≤ 0.05; **P ≤ 0.01; ***P ≤ 0.001; n.s., not significant (two-tailed non-parametric Mann–Whitney *U*-test). Source data are provided as a Source data file.

Consistent with the in vivo observation, we found no difference between conditioned medium from mature adipocytes isolated from HFD-fed wild-type and Ad-Piezo1-KO mice with regard to its effect on proliferation of 3T3-F442A preadipocytes or SVF cells (Fig. 5a). In contrast, conditioned medium from adipocytes from wild-type HFD-fed animals induced the differentiation of preadipocytes and SVF cells, whereas conditioned medium from Piezo1-deficient adipocytes or from adipocytes obtained from SD-fed mice had little effect on differentiation (Fig. 5a–c). This suggested that adipocytes from HFD-fed mice produce a transferrable factor in a Piezo1-dependent manner, that promotes the differentiation of adipocyte precursor cells. Several known adipogenic mediators including IGF1, FGF1, FGF2, Jag-1, TGFβ, adiponectin, BMP3, and WNT5a^17,18,32^ (Fig. 6a) as well as several lipids (Supplementary Table 1) were found to be released from mature wild-type adipocytes, but only the release of FGF1 was increased by the Piezo1 activator Yoda1 (Fig. 6a, and Supplementary Table 1), and FGF1 release was also increased by hypoosmotic membrane stress induced by short term exposure to a hypotonic solution in mouse and human adipocytes (Fig. 6b, c). Consistent with reports that FGF1 is released from cells in a non-classical manner by direct translocation through the plasma membrane^33^, Piezo1-dependent FGF1 release from adipocytes was not affected by the vesicle formation and transport inhibitor brefeldin A, but was blocked by chelation of intracellular Ca^2+^ using BAPTA-AM (Fig. 6d, e). In Piezo1-deficient adipocytes, FGF1 expression was unchanged (Supplementary Fig. 3a–c), but the basal FGF1 release, as well as the effect of Yoda1 and hypoosmotic membrane stress on FGF1 release, was clearly reduced (Fig. 6a, b and Supplementary Table 1). The ability of conditioned medium to induce preadipocyte differentiation was strongly decreased after depletion of FGF1 from the medium (Supplementary Fig. 2d, e), and the defect of conditioned medium from Piezo1-deficient adipocytes to induce preadipocyte differentiation was rescued by addition of FGF1 as indicated by an increased number of lipid-containing cells while the average lipid content per cell remained unchanged (Fig. 6f and Supplementary Fig. 3f, g). Antagonism of FGF receptor 1 (Fgfr1), the major FGF receptor expressed in preadipocytes (Supplementary Fig. 3h, i), with PD173074 significantly reduced preadipocyte differentiation in response to FGF1 and conditioned medium from mature wild-type adipocytes (Fig. 6f). To further test the involvement of FGFR1 in the regulation of preadipocyte differentiation we generated mice lacking FGFR1 in preadipocytes by crossing mice carrying a floxed Fgfr1 allele^34^ with Pdgfrα-CreERT2 animals^35–37^ (Pα-Fgfr1-KO; Supplementary Fig. 4a, b). Loss of FGFR1 in adipocyte precursor cells of the SVF strongly inhibited the ability of conditioned medium (CM) of adipocytes prepared from HFD-fed wild-type mice to induce adipocyte differentiation as indicated by strongly reduced lipid droplet formation (Fig. 6g). The SVF from induced PdgfrαCreERT2;Fgfr1^flox/flox^ also did not show an increase the expression of several adipocyte marker genes including Pparγ2 and C/ebpα when exposed to adipocyte CM from HFD fed mice or to FGF1 (Supplementary Fig. 4c, d). The FGF1-induced differentiation of SVF cells was reduced by the phosphatidylinositol 3-kinase (PI-3-K) inhibitor LY294002 as well as by the MAP-kinase kinase (MEK1/MEK2) inhibitor PD98059, whereas the p38 mitogen-activated protein kinase inhibitor SB253580 had no effect, and a combination of the PI-3-kinase and p38 kinase inhibitor blocked the effect of FGF1 (Supplementary Fig. 4d). Induced Pdgfrα-CreERT2;Fgfr1^flox/flox^ mice fed a HFD recapitulated the phenotype of Ad-Piezo1-KO animals with increased adipocyte size and reduced adipocyte number (Fig. 6h–j and Supplementary Fig. 4e, f). When we in vivo-labeled proliferating adipocyte precursors in WAT of induced Pdgfrα-CreERT2;Fgfr1^flox/flox^ mice with BrdU during the first week of HFD feeding, we found after 8 weeks a reduced differentiation of adipocyte precursors into mature adipocytes as indicated by the strongly reduced number of BrdU-positive adipocytes (Fig. 6k). In contrast, the number of BrdU-labeled PDGFRα-positive vWAT stromal cells did not differ between wild-type and Pdgfrα-CreERT2;Fgfr1^flox/flox^ mice after 1 week (Supplementary Fig. 4g).

## Discussion

The fact that loss of Piezo1 in mature adipocytes results in a reduced differentiation of adipocyte precursor cells under a HFD indicates that mature adipocytes generate an adipogenic signal which promotes adipocyte precursor differentiation. Among several candidates, we identified FGF1 as a mediator which is released from adipocytes upon Piezo1 activation and promoted adipocyte precursor differentiation. In line with our findings, FGF1 expression has been shown to increase in obese humans and mice^32,38^ and to promote preadipocyte differentiation^39,40^. The role of FGF1 as a mediator of Piezo1-dependent adipogenesis is also supported by the fact that the phenotype of adipocyte-specific Piezo1-deficient animals resembles that of FGF1-deficient mice, which upon feeding with HFD show an increased number of very large adipocyte, decreased insulin sensitivity, increased inflammation and a restriction of the phenotype to the visceral adipose tissue^32^. We also identified FGFR1 as the FGF-receptor subtype on preadipocytes which mediates the effect of FGF1. Our in vitro data indicate that this FGFR1-mediated effect involved PI-3-kinae/AKT and MEK/ERK signaling. Interestingly, in Pdgfrα-Cre;Akt2^flox/flox^ mice, loss of AKT2 in adipocyte precursor cells leads to reduced HFD-induced adipogenesis^15^ similar to the phenotype we observed in induced Pdgfrα-CreERT2;Fgfr1^flox/flox^ animals. This suggests that FGFR1 and AKT2 may function in the same pathway in preadipocytes.

Piezo1 can be activated by various mechanical stimuli acting on cellular membranes including mechanical indentation of the cell surface, cell migration, exposure to fluid shear stress, compression of the cell membrane, osmotic swelling or forces generated at the cell–cell or cell–matrix interface^41,42^. How Piezo1 is activated in hypertrophic adipocytes is not known. According to the law of Laplace, it is conceivable that the increase in adipocyte volume per se might result in an increase in plasma membrane tension sufficient to activate Piezo1. In fact, we show that membrane tension in adipocytes increases already after 7 days of HFD feeding. In addition, compressive forces or strain exerted by the extracellular matrix on expanding adipocytes have been described^43,44^ and may also be able to induce Piezo1 activation. Strikingly, native adipocyte Piezo1 currents lacked the classical fast inactivation^28^, a phenomenon described in other cells before^45^. While the mechanism of slow inactivation kinetics remains unknown, it indicates that adipocyte Piezo1 might operate in response to chronic mechanical stress as expected for hypertrophic adipocytes.

Recently, evidence was provided that the volume-regulated anion channel (VRAC) is activated by an increase in adipocyte volume during adipocyte hypertrophy^46^. In contrast to the role of Piezo1 in visceral adipocytes described here, VRAC activation rather promotes adipocyte expansion and insulin signaling, and loss of VRAC results in reduced adipocyte size under HFD^46^. While VRAC thereby mediates a feed-forward mechanism to promote adipocyte expansion and to enhance insulin signaling during increased caloric intake, Piezo1 rather functions as a feed-back regulator which promotes adipogenesis under a hypercaloric diet via FGF1 and FGFR1. Thus, VRAC and Piezo1 both appear to be part of a complex regulatory system by which mature adipocytes control WAT homeostasis during obesity.

A consequence of induced adipocyte-specific loss of Piezo1 expression was a reduced glucose tolerance and insulin sensitivity as well as an increased inflammatory activity in the visceral adipose tissue under HFD, a phenotype also seen in constitutive adipocyte-specific Piezo1-deficient mice^47^. In contrast to our findings in acutely induced adipocyte-specific Piezo1-deficient mice, constitutive Piezo1-deficient mice had no significantly reduced adipocyte size but showed an increase in liver triglyceride content^47^. This may be the consequence of compensatory changes induced by long-term loss of Piezo1 in the constitutive knock-out model. A reduction in glucose tolerance and insulin sensitivity as well as increased adipose tissue inflammation were also observed in other mouse models with increased adipocyte cell size and decreased adipocyte hyperplasia^32^ whereas animal models with reduced adipocyte cell size and increased hyperplasia show improved insulin sensitivity and reduced adipose tissue inflammation^48,49^. Similar correlations between adipogenesis, adipocyte cell size, and metabolic consequences have been observed in humans^30,31^. Although the precise mechanisms linking adipocyte hypertrophy to inflammation and insulin resistance are incompletely understood, hypoxia in the hypertrophic adipose tissue has been shown to be an important mediator of hypertrophy-induced inflammation and insulin resistance^50–53^.

In this study, we show that the mechanosensitive cation Piezo1 on mature adipocytes is a critical mediator of hypercaloric diet induced adipocyte hyperplasia. This discovery identifies a mechanism that is intrinsic to the WAT and increases adipocyte cell number in response to a positive energy balance. Our findings strongly support the concept that mechanical force is a major determinant of obesity and may help to identify novel therapeutic approaches to prevent or treat pathological obesity and associated disorders.

## Methods

### Cells

For the isolation of human and mouse adipocyte as well as SVF, adipose tissue was digested with collagenase II (0.25 mg/ml, Sigma) in a buffer containing 125 mM NaCl, 5 mM KCl, 1 mM CaCl_2_, 2.5 mM MgCl_2_, 1 mM KH_2_PO_4_, 2% BSA, 4 mM glucose, and 25 mM Tris (pH 7.4) for 1 hour at 37 °C. Cells were filtered through a nylon mesh, and the cell suspension including adipocytes and the SVF was washed 3 times with the same buffer while keeping the floating adipocytes untouched. Thereafter, adipocytes were harvested. The wash buffer was filtered through a 40 μm filter and was centrifuged for 30 min at 350 × *g* to collect the SVF. Isolated adipocytes and SVF were cultured at 37 °C and 5% CO_2_ in DMEM with 10% FBS and 100 U/ml penicillin and streptomycin each.

To prepare conditioned medium from primary adipocytes for SVF and 3T3F442A in vitro differentiation assays, the adipocyte supernatant was collected after 16 h of culture, centrifuged and then filtered through a 0.22 μm filter (Millipore). As a control for experiments using the conditioned medium, SVF cells was either kept in non-conditioned medium or were kept 2 days after reaching confluency in DMEM containing 1 μg/ml insulin (Gibco), 0.25 μg/ml dexamethasone (Sigma), 30 μg/ml 3-isobutyl-1-methylxanthine (Sigma) to induce differentiation of adipocyte precursor cells. Conditioned medium was given to the SVF 2 and 4 days after cells had reached confluency. Thereafter cells were cultured for an additional 6 days in DMEM supplemented 5 μg/ml insulin with medium changes every 2 days. 3T3-F442A cells were obtained from the European Collection of Authenticated Cell Cultures (ECACC), and cells were grown in DMEM with 10% FBS and 100 U/ml penicillin and streptomycin each. As a control for experiments using conditioned medium, 3T3-F442A cells were kept in the non-conditioned medium. To induce differentiation, 3T3-F442A cells were cultured in DMEM plus 10% FBS and 5 μg/ml insulin after reaching confluency for 2 days. Conditioned medium was given to 3T3-F442A cells 2 and 4 days after cells had reached confluency. Thereafter cells were cultured for 4 days in DMEM with 10% FBS. In some experiments, the SVF was incubated with 10 nM FGF1 (R&D) without or with 10 μM LY294002 (Sigma), 10 μM SB253580 (Enzo Life Science) or 10 μM PD98059 (Sigma) for 2 days, and the expression of adipocyte marker genes Pparγ2 and C/ebpα was determined.

To prepare conditioned medium for adipogenic mediator and lipid measurment, freshly isolated primary adipocytes were washed and then cultured for 16 h in DMEM with 10% FBS and 100 U/ml penicillin and streptomycin. This basal conditioned medium was harvested, centrifuged at 1000 × *g* for 10 min at 4 °C to remove any cell debris, and filtered through a 0.22 μm filter (Millipore) for Elisa and LC-MS/MS analysis. To immunodeplete FGF1 from adipocyte-conditioned medium, 2 μg of monoclonal anti-FGF1 antibody (Santa Cruz, # sc-55520) or control IgG (Santa Cruz, # sc-3879) were added to 200–300 μl of adipocyte-conditioned medium filtrates and incubated for 15 h at 4 °C under gentle agitation. 60 μl of protein G-coupled agarose beads (Santa Cruz) were added to the solution and gently mixed, followed by incubation for 6 h at 4 °C under gentle agitation. Supernatants were collected and filtered after centrifugation at 300 × *g* for 30 s and used for differentiation experiments^54^.

To measure Yoda1 induced release of adipogenic mediators and lipids, freshly isolated primary adipocyte were cultured for 16 h in DMEM with 10% FBS and 100 U/ml penicillin and streptomycin. Cells were then gently washed 5 times with HBSS supplemented with 2% fatty acid–free BSA. Thereafter, the medium was replaced by fresh HBSS containing Yoda1 or carrier solution (0.1% DMSO) for the indicated time periods. This medium was then harvested, centrifuged, and filtered for Elisa and LC-MS/MS analysis as described above. To induce hypotonic membrane stress, primary adipocytes were exposed to hypotonic buffer contained (in mM): 90 NaCl, 2 CsCl, 1 MgCl_2_, 1 CaCl_2_, 10 HEPES, 10 mannitol, pH 7.4 with NaOH (210 mOsm kg^−1^). Isotonic extracellular solution had the same composition, but contained 110 mM instead of 10 mM mannitol (300 mOsm kg^−1^)^46^.

### Oil-Red-O and BODIPY staining

Differentiated SVF and 3T3-F442A cells were fixed with 2% formaldehyde and 0.2% glutaraldehyde in PBS for 15 min and then washed 3 times in PBS for 10 min. For Oil-Red-O staining, cells were washed for 30 s in 60% isopropanol and stained with Oil-Red-O (0.7% in 60% isopropanol; Sigma) for 1 h and rinsed with 60% isopropanol for 1 min followed by water. Oil-Red-O stained cells were directly imaged using an inverted microscope or lipid content was quantified after Oil-Red-O extraction with isopropanol by determination of light absorbance at 490 nm. For BODIPY staining, fixed cells were blocked in 2% BSA in PBS with 0.3% Triton X-100 for 1 h and incubated with BODIPY (Invitrogen, catalog number D3922, 1:500), DAPI (Invitrogen, catalog number D1306, 1:1000) and anti-Ki67 antibodies (Abcam, catalog number ab15580, 1:200) for 1 h at room temperature. Cells were washed and incubated with donkey anti-rabbit AF-594 (Molecular Probes #A32754, 1:500) secondary antibody for 1 h and washed with PBS, followed by imaging with Leica TCS SP5 confocal microscope.

### siRNA-mediated knock-down

At density of 1–2 × 10^4^/cm^2^, cells were transfected with siRNAs using Opti-MEM and Lipofectamine RNAiMAX (Invitrogen)^55^. SiRNAs were from Qiagen or Sigma. 50 pmoles siRNA were mixed gently with RNAiMAX in 100 μl Opti-MEM and incubated for 30 min at room temperature and added to 1.5 ml cell culture medium (DMEM). Cells were incubated with the complexes for 6 h at 37 °C in a CO_2_ incubator and thereafter medium was replaced by complete medium (DMEM plus 10% FBS). The transfection was repeated next day and assays were performed 48 h after the second transfection. The targeted sequences of siRNAs directed against RNAs encoding PIEZO1 were 5′-CACCGGCATCTACGTCAAATA-3′ and 5′-TCGGCGCTTGCTAGAACTTCA-3′.

### Determination of membrane tension in primary adipocytes

Primary visceral adipocytes from mice fed with standard diet or HFD for 7 days were isolated and incubated with 1 μM of the membrane tension probe Flipper-TR® (Tebu-bio, #SC020) for 30 min at 37 °C as described^29^. The adipocytes were then washed 3 times with HBSS and imaged with a Leica-SP8 FLIM microscope. Excitation was performed using a pulsed 488 nm laser (Laser kit WLL2+pulse picker, Leica Microsystems) operating at 80 MHz, and the emission signal was collected from 549 to 651 nm with acousto-optical beam splitter (AOBS) using a gated hybrid (HyD SMD) detectors and a TimeHarp 300 TCSPC Module and Picosecond Event Timer (PicoQuant). SymPhoTime 64 software (PicoQuant) was then used to fit fluorescence decay data. To extract lifetime information, the photon histograms from membrane regions were fitted with a double exponential, and 2 fluorescence emission decay times (τ1 and τ2) are extracted. The longest lifetime with the higher fit amplitude τ1 is used to report membrane tension^29^.

### Determination of [Ca^2+^]_i_

For the determination of the intracellular Ca^2+^ concentration, 3T3-F442A cells and adipocytes were loaded with 5 μM Fluo-4 AM (Molecular Probes, #F14201) for 1 h and attached with 0.025% polyethyleneimine (Sigma) for 30 min on a glass imaging plate from Eppendorf. Live-cell images were acquired with an Olympus IX81 microscope. Fluorescent intensity was measured with a FlexStation 3 (Molecular Devices).

### Electrophysiology

Cell attached patch-clamp recordings (borosilicate pipette resistance of 1.2–1.4 Mohms) were performed as previously described^56,57^. Briefly, currents were elicited by a HSPC1 high speed pressure clamp (Ala, USA) at various holding potentials, as indicated. The pipette solution was: 150 NaCl, 5 KCl, 1 Mg^2+^, 2 Ca^2+^, 10 Hepes, pH: 7.35. The bath solution contained: 150 KCl, 3 Mg^2+^, 10 Hepes, 5 EGTA, pH: 7.2. Currents were recorded with a Multiclamp 700B patch clamp amplifer (Axon Instruments, USA), filtered at 1 kHz and digitized at 10 kHz using Clampex (Axon Instruments, USA).

### Immunoblotting

Cells and tissues were lysed in RIPA buffer (Cell Signaling Technology, #9806) supplemented with protease and phosphatase inhibitors (Cell Signaling Technology, #5872). Lysates were centrifuged at 10,000 × *g* for 10 min then subjected to SDS-PAGE and transferred to nitrocellulose membranes. Membranes were probed with primary and HRP-conjugated secondary antibodies (Cell Signaling Technology) and were developed using the ECL detection system (Pierce). Anti-Piezo1-antibody was from Proteintech (catalog number 15939-1-AP, 1:200), anti-GAPDH, anti-FGFR1 and anti-AKT antibodies were from Cell Signaling Technology (#2188,#9740 and #9272, respectively, 1:500) and anti-FGF1 antibody was from Santa Cruz Biotechnology (sc-55520, 1:200).

### Animal models

All mice were back-crossed onto a C57BL/6N background at least 8–10 times, and experiments were performed with littermates as controls. For in vivo experiments, male animals were used at an age of 8–12 weeks if not stated otherwise, while adipose tissue from female and male mice were tested in parallel in several in vitro experiments as indicated. Mice were housed under a 12-h light–dark cycle with free access to food and water and under specific pathogen-free conditions if not stated otherwise. Mice carrying a floxed allele of the gene encoding Piezo1^26^ and Fgfr1^34^ as well as the Cre reporter line Gt(ROSA)26Sor^tm4(ACTB-tdTomato,-EGFP)Luo^/J (mT/mG)^58^ were obtained from The Jackson Laboratory. Animals expressing the lacZ gene under the control of the Piezo1 promoter were obtained from the Knockout Mouse Project (KOMP). Mice that allow for tamoxifen-dependent adipocyte-specific Cre activation have been described before^59^ and Pdgfrα-CreERT2 animals^35^ were provided by the RIKEN BRC through the National BioResource Project of the MEXT/AMED, Japan (RBRC09616). To induce recombination, animals were treated with 5 ×1 mg/d tamoxifen (Sigma) dissolved in corn oil, and 7–14 days later experiments were started. All animals which served as controls for tamoxifen-induced adipocyte-specific animals were treated with the same amount of tamoxifen under the same conditions. Mice were fed a standard pellet chow (Altromin) or a HFD containing 30% (w/w) fat (ssniff) with water ad libitum.

### Calorimetry

Mice were placed into the analysis cages of the PhenoMaster-System (TSE-Systems, Bad Homburg, Germany) for 48 h. Following 48 h of acclimatization, O_2_ consumption and CO_2_ production were measured every 10 min for a total of 48 h and converted to kcal/h using the Weir equation. Data were analyzed using the PhenoMaster-Software (TSE-Systems, Bad Homburg, Germany).

### Glucose and insulin tolerance test

After overnight fasting glucose was administered to mice intraperitoneally at a dose of 2 mg/g body weight when on SD and of 2.75 mg/g body weight when on HFD. Blood samples were taken from the tail vein before and after glucose application at indicated time points, and glucose levels were measured using a glucometer (Accu-Chek Aviva). For insulin tolerance test, mice were injected intraperitoneally with insulin (0.75 mU/g), and glucose levels were determined as described above.

### Histology, immunohistochemistry, TUNEL assay

H&E staining was performed on 4-μm paraffin sections according to standard protocols. For immunohistochemical staining of adipose tissue sections, 12-μm cryosections were treated according to the instructions of the In Situ Cell Death Detection Kit (Roche Applied Sciences). After washing twice in PBS, slides incubated for 10 min with DAPI (Invitrogen), and finally mounted in Mowiol (Calbiochem/EMD Biosciences) and analyzed on a Leica TCS SP5 confocal microscope by an experimenter blinded to genotype. Using the ImageJ software adipocyte volume was calculated from H&E stained paraffin sections as describes previously^60^. Adipocyte number was calculated by dividing epididymal adipose tissue volume, calculated by multiplying epididymal adipose tissue weight and commonly used adipose tissue density factor 0.92 g/cm^3^, by adipocyte volume^61^.

### BrdU and EdU labeling

For BrdU in vivo proliferation assay, mice were supplied with water containing 1 mg/ml BrdU (Merck; catalog number 203806) for 1 week. In parallel, feeding of mice with HFD was initiated. After 1 or 8 weeks, adipose tissue was prepared, minced, and fixed in zinc formaldehyde for 24 h at 4 °C. The samples were then washed and blocked with 2% BSA in PBS and stained overnight at 4 °C with antibodies including anti-Perilipin-1 (Abcam # ab61682, 1:500), anti-BrdU (Abcam # ab6326, 1:300), anti-PDGFRα (R&D #AF1062, 1:200). The samples were then washed with PBS three times for 10 min at room temperature. Secondary antibodies including goat anti-mouse AF-546, (Molecular Probes # A11003, 1:500), donkey anti-rabbit AF-488 (Molecular Probes #A11008, 1:500), rabbit anti-goat AF-594 (Molecular Probes #A11080, 1:500) were applied in PBS with 2% BSA and 0.3% Triton X-100. Some tissues were further incubated with DAPI (Invitrogen), LipidTOX™ (Molecular Probes #H34475) for neutral lipid staining and CellMask Orange (Molecular Probes #C10045, 1:400) for plasma membrane stain for 1 h at room temperature, followed by three washes with PBS at room temperature in the dark. The tissues were then imaged with a Leica confocal SP5 microscope. Adipocyte nuclei were identified by their location between plasma membrane and adipocyte lipid droplets as described by Jeffery et al.^15^. Nuclei that are located between two plasma membranes are non-adipocyte nuclei and nuclei that are not separated from the lipid droplets by a plasma membrane are the mature adipocyte nuclei.

For EdU in vivo incorporation assay, mice were supplied with water containing 500 μg/ml EdU (Lumiprobe; catalog number 40540) for 1 week. In parallel, feeding of mice with HFD was initiated. After 1 or 8 weeks, adipocytes and SVF from epididymal WAT were isolated as described above.

### Caspase-3 activity assay

White adipose tissue or isolated adipocytes were homogenized in a dounce homogenizer in lysis buffer (10 mM Tris-HCl, 10 mM Na_2_HPO_4_/NaH_2_PO_4_, pH 7.5, 130 mM NaCl, 1% Triton -X-100, 10 mM NaPPi) and kept on ice for 30 min. Samples were centrifuged for 15 min at 4 °C and 18.400 × *g*, and supernatants taken for caspase-3 activity assay and protein concentration determination. For caspase-3-activity assays 20 µl sample and 200 µl assay buffer (20 mM HEPES (pH 7.5), 10% glycerol, 2 mM DTT, 20 μM Ac-DEVD-AMC) were incubated at 37 °C for 2 h and luminescence was measured (FlexStation 3, Molecular Devices).

### Adipocyte pulse-chase experiments

For adipocyte pulse-chase experiments, mice were treated with 5 ×1 mg/d tamoxifen by intraperitoneal injection for 5 consecutive days starting at 8 weeks of age, and were then allowed to recover for 1 week. Mice were then placed on HFD or remained on SD for a 1- or 8-week chase period. Inguinal and epididymal WAT were taken from several regions throughout the depot, and analyzed by whole-mount confocal microscopy for tdTomato and eGFP expression. For each data point, at least 300 (sWAT) or 2000 (vWAT) adipocytes were counted from multiple images from each depot of each animal.

### Flow cytometry and cell sorting

For adipocyte sorting and size distribution analysis, primary mouse adipocytes were freshly isolated from inguinal or epididymal fat pads as described above and then stained with Hoechst 34580 (392/440 nm, Invitrogen #H21486, 1:500) and Calcien-AM (495/515 nm, BD #564061, 1:500). In experiments involving mT/mG reporter mice, adipocytes were only stained with Hoechst. The cell suspension was then loaded into the sample cartridge and analyzed or sorted using a BioSorter large particle flow cytometer (Union Biometrica) equipped with a 500 μm metal-free FOCA (Fluidics and Optics Core Assembly). Physical characteristics of size (TOF), optical density (EXT), and three different channels of fluorescence signals (FLU) were collected. Adipocytes were carried through the flow cell, one by one, and passed through the focus of a laser beam. Relative size was determined by the time of flight (TOF) measurement and calibrated with megabead particle size standards (Polysciences). The optical density of the object was determined by the extinction (EXT) measurement. Spatial information on fluorescence and extinction for each adipocyte passing through the flow cell were collected by using Profiler II™. Solid state 405 nm and 488 nm lasers were used in the experiments. Calcein-AM or GFP fluorescence was collected using BP510/23 nm filter (PMT2) and Hoechst fluorescence using BP 440/30 nm (PMT1).

For analysis of EdU incorporation, adipocyte nuclei was prepared by lysing adipocytes for 5 min in 0.2% IGEPAL in HBSS with vortexing every 1–2 min and centrifugation at 3000 × *g* for 10 min. SVF was first stained with extracellular antibodies including anti-PDGFRα (APC, eBioscience #17-1401), anti-CD34 (AF700, eBioscience #57-0341), anti-CD31 (efluor450, eBioscience #48-0311-80) and anti-CD45 (efluor450, eBioscience #48-0451-80, 1:100) antibodies for 30 min on ice, and then washed with PBS. Both adipocyte nuclei and SVF were fixed in Fix/Perm (eBioscience #00-512343) and permeabilized with permeabilization buffer (eBioscience #00833356). Samples were then treated with 6000 unit/ml DNase (Fisher Scientific) in PBS for 2 h and processed for propidium Iodide (Invitrogen R37169) staining and EdU staining using Edu Alexa Fluor™ 488 or 647 Flow Cytometry Assay Kit (Invitrogen #C10632 and #C10634). Flow cytometry data were collected on BD FACS Canto II (Becton Dickinson) and analyzed using the FlowJo software package. For assessment of lipid positive cell numbers after SVF differentiation, SVF cells differentiated in 6-well plate were trypsinized, fixed in Fix/Perm (eBioscience #00-512343) and permeabilized with permeabilization buffer (eBioscience #00833356). Half of cells were then stained for BODIPY (Invitrogen, catalog number D3922). BODIPY was excited with a 488 nm green laser and emission was recorded at 503 nm using a BD FACS Canto II (Becton Dickinson). The rest of the cells were stained for Oil Red O as described above to calculate lipid content.

### Plasma levels of insulin and IL-6

Blood samples were collected from the tail vein or the retro-orbital plexus in EDTA (10 mM final concentration). Plasma IL-6 and insulin levels were determined using the MAGPIX system combined with MILLIPLEX MAP magnetic bead–based multi-analyte panels and MILLIPLEX Analyst 5.1 software (Merck Millipore).

### Determination of adipogenic mediators and lipids

Concentrations of IGF-1, FGF-1, FGF-2, Jag-1, TGF-β, BMP3, adiponectin, WNT-5a and WNT10B in supernatants of adipocytes were determined using ELISA kits. IGF-1 and TGF-β ELISAs were from Bio-Techne GmbH. FGF-1 and BMP2 ELISA was from Abcam, adiponectin ELISA kit was from Millipore and FGF-2, Jag-1, BMP3, WNT-5a, and WNT10B ELISAs were from antibodies-online GmbH.

For lipid measurement, conditioned medium (500 μl) was spiked with 10 μl internal standard stock solution which containing 22 deuterium labeled oxylipin standards and ^13^C-labeled arachidonic acid. Lipids were extracted using a two-phase liquid–liquid extraction in which ethyl acetate (1000 μl) was added, samples were rigorous vortexed, centrifuged (10,000 × *g*, 5 min, 4 °C), and the upper organic phase was collected. The extraction was repeated one more time. These lipid extracts were evaporated to dryness under a continuous nitrogen stream (Vacuum manifold, Macherey-Nagel, Düren, Germany) and subsequently resuspended in methanol/water (1:1). 8 μl of each sample were analyzed by UPLCMS/MS for primary fatty acids and oxylipins. The polyunsaturated fatty acid (PUFA)-derived lipid mediators were detected using a custom LC-MS/MS screening library that was developed to assess the involvement of such mediators in inflammation, angiogenesis, and metabolic diseases. The assay used detects 112 unique lipid species, derived from cyclooxygenase, lipoxygenase, and cytochrome P450 metabolism. The UPLC-MS/MS analyses were performed as described (11), with minor modifications. Reversed-phase separation was performed on an Acquity UPLC BEH shield RP18 column (2.1 × 100 mm; 1.7 μm; Waters, Milford, USA) on an Agilent 1290 Infinity LC5 system (Agilent, Waldbronn, Germany). The mobile phase consisted of (A) ACN/water/acetic acid (60/40/0.02, v/v/v) and (B) ACN/isopropanol (50/50, v/v). Elution of analytes was carried out for 5.8 min at a flow rate of 0.5 mL/min. Gradient conditions were as follows: 0–4.5 min, 0.1–55% B; 4.5–5.0 min, 55–99% B; 5.0–5.8 min, 99% B, followed by are-equilibration step 0.1% B for 2 min. Mass spectrometry was performed on a QTrap 5500 mass spectrometer (Sciex, Darmstadt, Germany), equipped with a Turbo V ion source. Electro spray ionization in negative mode was employed. The ion source parameter were as followed, CUR 20 psi, IS −4500 V, TEM 525 °C, GS1 30 psi, GS2 30 psi, CAD medium (nitrogen was employed as the collision gas). Analyst 1.6.2 and MultiQuant 3.0 (Sciex, Darmstadt, Germany), were used for data acquisition and analysis, respectively.

### Expression analysis

RNA isolation and transcription was performed as described previously^55^. Total RNA was isolated from cells using the RNeasy mini or micro kit (Qiagen) according to the manufacturer’s protocol. For fat tissues, RNeasy lipid tissue Mini Kit (Qiagen) was used to isolate total RNA. DNA was removed using the QIAGEN RNase-Free DNaseSet. Complementary DNA synthesis was performed using the ProtoScript II Reverse Transcription kit (New England BioLabs, M0368S). Primers were designed with the online tool provided by Roche and quantification was performed using the LightCycler 480 Probe Master System (Roche). Genomic DNA was used as a universal standard to calculate gene copy number per nanogram of RNA. Relative expression levels were obtained by normalization with GAPDH or 18S values.

### qPCR primer sequences

CD11b 5′-tctggcagatgtggctattg-3′ and 5′-gtcctgtcttgaggctccat-3′;

CD68 5′-ttctgctgtggaaatgcaag-3′ and 5′-agagagagcaggtcaaggtga-3′;

Nos2 5′-aaggggacgaactcagtgg-3′ and 5′-cccggaaggtttgtacagc-3′;

Arg1 5′-ggcctttgttgatgtcccta-3′ and 5′-acagaccgtgggttcttcac-3′;

CD137 5′-caagagctgccctccaagta-3′ and 5′-cagatgttacagttcggctgtc-3′;

CD11c 5′-gagccagaacttcccaactg and 5′-tcaggaacacgatgtcttgg-3′;

HOXC1 5′-gcagcaagcacaaagagga-3′ and 5′-cgtctggtacttggtgtaggg-3′;

F4/80 5′-tctctaaactcaaggacacgaggt-3′ and 5′-ctggaaaatgcccagcac-3′;

IL6 5′-tgatggatgctaccaaactgg-3′ and 5′-ttcatgtactccaggtagctatg-3′;

IL1A 5′-ttggttaaatgacctgcaaca-3′ and 5′-gagcgctcacgaacagttg-3′;

MRC2 5′-ccccaaactccgacactg-3′ and 5′-gggcctggatccaactct-3′;

TNFα 5′-tcttctcattcctgcttgtgg-3′ and 5′-ggtctgggccatagaactga-3′;

Adipoq 5′-cctaccttccttccagactgtgt-3′ and 5′-agaagagaaagaaacccagcaa-3′;

Perilipin 5′-ggacttacaaacagcaacagacc-3′ and 5′-catctgtgagttggtggacact-3′;

PPARγ1 5′-acgcgaggaggtcaagaag-3′ and 5′-tcaatgggagttaagaagaattt-3′;

PPARγ2 5′-tctgtgtcaaccatggtaatttc-3′ and 5′-tgctgttatgggtgaaactctg-3′;

Cebpα 5′-cgctggtgatcaaacaagag-3′ and 5′-ggtggctggtaggggaag-3′;

FABP4 5′-caggctcagactcagcagttt-3′ and 5′-cacagggaactgagcagtga-3′;

FAS 5′-gcaggcagtcagtcagca-3′ and 5′-aggctttctcccttagggtct-3′;

CD36 5′-ttgaaaagtctcggacattgag-3′ and 5′-tcagatccgaacacagcgta-3′;

FGFR1 5′-ttgaaagattatgttttggttctctg-3′ and 5′-tggtgaactgtcttgtatccttaaac-3′;

FGFR2 5′-tgatagcagggtcctctcgt-3′ and 5′-caaggcaaacgtgatgaatg-3′;

FGFR3 5′-ctgagaacgtgcgtctgg-3′ and 5′-tgactaggagagccattcaagc-3′;

FGFR4 5′-cctgagatcagctggaagga-3′ and 5′-tcccctgaaagatgctcaac-3′;

FGF1 5′-cctgccagttcttcagtgc and 5′-ggctgcgaaggttgtgat-3′;

Piezo1 (human) 5′-cgtcttcgtggagcagatg and 5′-gcccttgacggtgcatac-3′;

Piezo2 (human) 5′-cactcagaaaatgcaacagca-3′ and 5′-ttccctctcttttgctcctaga-3′;

Piezo1 (mouse) 5′-ggaaaagagctccgacacac-3′ and 5′-ccaggacttccccacctatt-3′;

Piezo2 (mouse) 5′-agatcaagatgggcaacagg-3′ and 5′-ttcgtgattatcctgaaccaca-3′

### Other materials

Insulin was from Gibco. Yoda1 was from Maybridge (catalog number SPB07298). BAPTA-AM (catalog number B1205) was from Invitrogen. PD173074 (catalog number P2499) and brefeldin A (catalog number B5936) were from Sigma. Recombinant FGF1 was from R&D (catalog number 4686-FA). Anti-CD68 antibody was from Bio-Rad (catalog number MCA1957).

### Statistics

Trial experiments or experiments done previously were used to determine sample size with adequate statistical power. Samples were excluded in cases where RNA/cDNA quality or tissue quality after processing was poor (below commonly accepted standards). Animals were excluded from experiments if they showed any signs of sickness. The investigator was blinded to the group allocation and during the experiment. Data represent biological replicates. In all studies, comparison of mean values was conducted with unpaired, two-tailed Student’s t-test or one-way or two-way ANOVA with Bonferroni’s post hoc test. In all analyses, statistical significance was determined at the 5% level (*P* < 0.05). Depicted are mean values ± s.d. or ± s.e.m. as indicated in the figure legends. Statistical analysis was performed with Prism5 or Prism6 (GraphPad) or Excel (Microsoft) software.

### Study approval

The work on human adipocyte samples was approved by the ethical committee of Xi’an Jiaotong University (XJTU2018-249 and XJTU2019-12) and conforms to the guidelines of the 2000 Helsinki declaration. Written informed consent was obtained from all subjects before their participation. All procedures of animal care and use in this study were approved by the local animal welfare authorities and committees (Regierungspräsidium Darmstadt, Germany and Ethical Committee of Xi’an Jiaotong University, China).

### Reporting summary

Further information on research design is available in the Nature Research Reporting Summary linked to this article.

## Supplementary information

Supplementary Information

Reporting Summary

## Data Availability

The source data underlying Figs. 1b, d–f, 2a, b,d–l, 3a–c and e, 4b, c, e–h, 5a–c, 6a–g and i–k and Supplementary Figs. 1a, e–l, 2a–f, 3a, c, e–i, 4a–g are provided as Source Data file. All data are available from the corresponding authors upon request.

## Source data

Source Data
