# Author Correction: Adipocyte Piezo1 mediates obesogenic adipogenesis through the FGF1/FGFR1 signaling pathway in mice

**DOI:** 10.1038/s41467-022-31816-0

**Published:** 2022-07-13

**Authors:** ShengPeng Wang, Shuang Cao, Malika Arhatte, Dahui Li, Yue Shi, Sabrina Kurz, Jiong Hu, Lei Wang, Jingchen Shao, Ann Atzberger, Zheng Wang, Changhe Wang, Weijin Zang, Ingrid Fleming, Nina Wettschureck, Eric Honoré, Stefan Offermanns

**Affiliations:** 1 Department of Pharmacology, Max Planck Institute for Heart and Lung Research, Ludwigstr. 43, 61231 Bad Nauheim, Germany; 2 Cardiovascular Research Center, School of Basic Medical Sciences, Xi’an Jiaotong University Health Science Center, Key Laboratory of Environment and Genes Related to Diseases, No.76 West Yanta Road, Yanta District, Xi’an, China; 3 Université Côte d’Azur, Centre National de la Recherche Scientifique, Institut de Pharmacologie Moléculaire et Cellulaire, Labex ICST, Valbonne, France; 4 Department of Pharmacology and Pharmacy, The State Key Laboratory of Pharmaceutical Biotechnology, The University of Hong Kong, Hong Kong SAR, China; 5 Institute for Vascular Signalling, Centre for Molecular Medicine, Goethe University, Frankfurt am Main, Germany; 6 Max Planck Institute for Heart and Lung Research, Flow Cytometry Service Group, Ludwigstr. 43, 61231 Bad Nauheim, Germany; 7 Department of Hepatobiliary Surgery, First Affiliated Hospital of Xi’an Jiaotong University, Xi’an, China; 8 Center for Mitochondrial Biology and Medicine, School of Life Science and Technology, Xi’an Jiaotong University, Xi’an, China; 9 Department of Pharmacology, School of Basic Medical Sciences, Xi’an Jiaotong University Health Science Center, Xi’an, China; 10 Center for Molecular Medicine, Goethe University Frankfurt, Theodor-Stern-Kai 7, 60590 Frankfurt, Germany

**Keywords:** Fat metabolism, Metabolic disorders

Correction to: *Nature Communications* 10.1038/s41467-020-16026-w, published online 08 May 2020.

This article contains an error in Fig. 5b, in which the image showing stromal vascular fraction (SVF) exposed to conditioned medium of adipocytes from knock-out mice fed standard diet (KO-SD) is also presented to illustrate the condition in which SVF was exposed to conditioned medium of adipocytes from wild-type mice fed standard diet (WT-SD). The statistical analysis of the experiment shown in the bar diagram of Fig. 5b is not affected by this error, because it is not based on the analysis of images but on the photometric quantification of Oil-Red-O extracted from samples. The corrected version of Fig. 5 is:


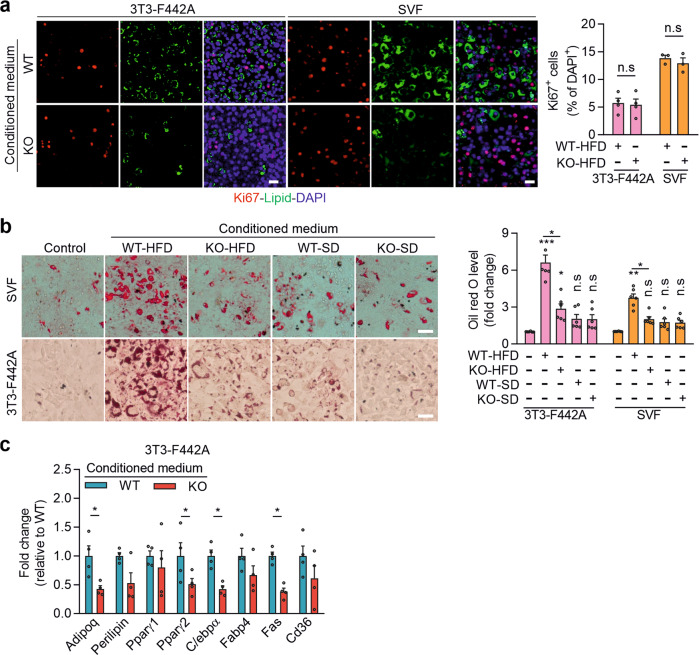


This replaces the previously incorrect version:
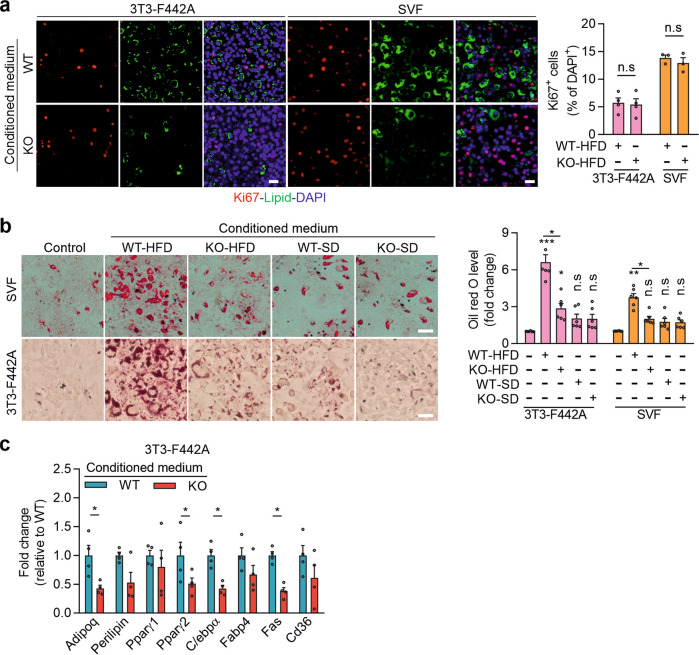


This has been corrected in both the HTML and PDF versions of the article.

