# Supplementary material for: Adipocyte Piezo1 mediates obesogenic adipogenesis through the FGF1/FGFR1 signaling pathway in mice
Source: Nat Commun. 2020 May 8;11:2303. doi: 10.1038/s41467-020-16026-w (PMC7211025; doi:10.1038/s41467-020-16026-w)
Supplement: Supplementary file 1 — Supplementary Information [file 41467_2020_16026_MOESM1_ESM.pdf]

Supplementary Information

Adipocyte Piezo1 mediates obesogenic adipogenesis through the FGF1/FGFR1 signaling pathway in mice

(Wang et al.)

Suppl. Figure 1

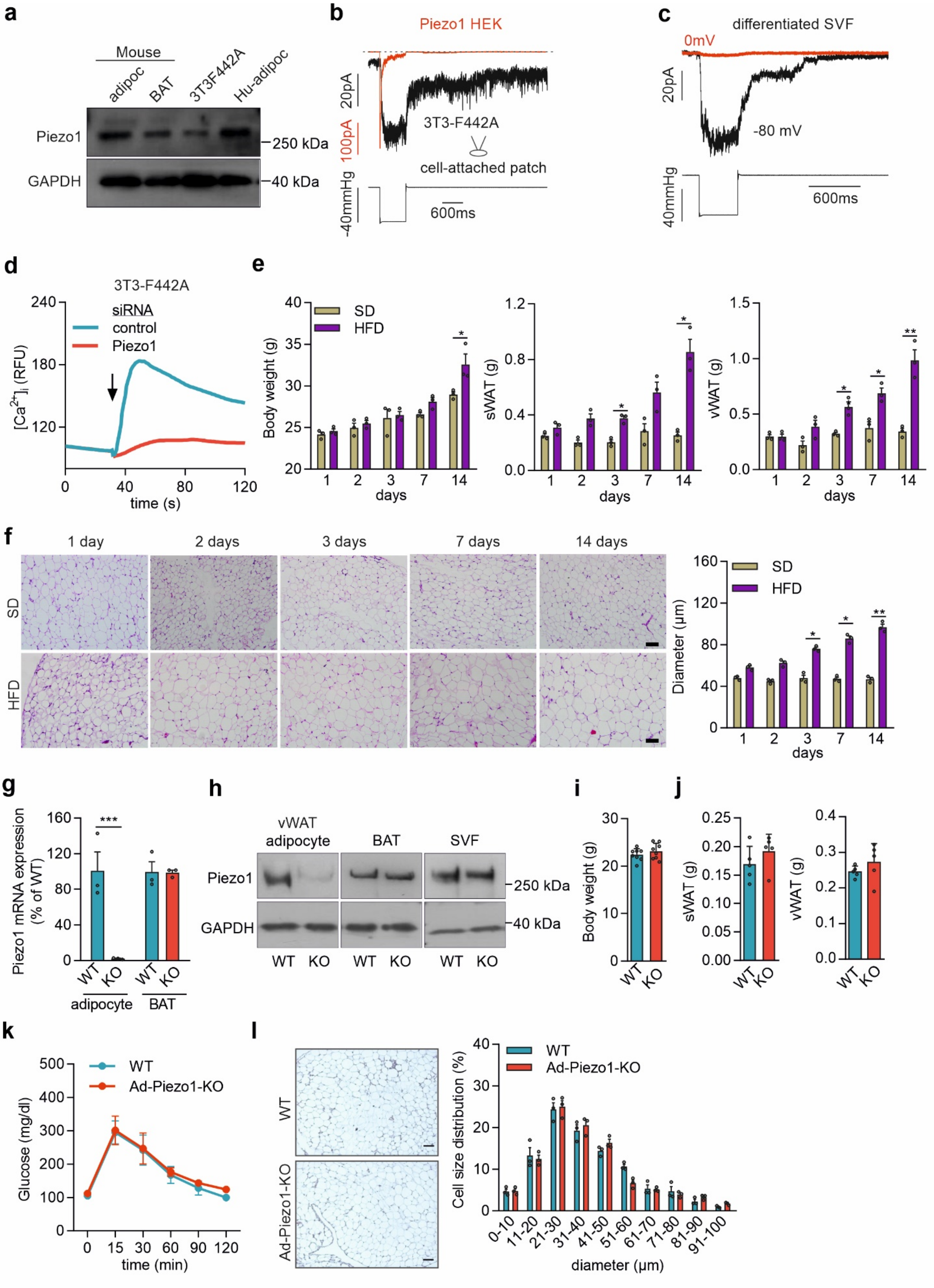

Wang et al., Suppl. Fig. 1

Suppl. Fig. 1. Phenotype of Ad-Piezo1-KO mice under SD.

(**a**) Immunoblot showing expression of Piezo1 in adipocyte from mouse vWAT (adipoc.), human vWAT (human-adipoc.), in BAT and in 3T3F442A cells. Shown is a representative of 3 independent experiments. (**b**) Inactivation kinetics of Piezo1 in 3T3-F442A cells and in transfected HEK cells (cell-attached patch clamp configuration). The holding potential was -80 mV and pressure pulses were applied every 10 seconds. (**c**) Recordings of stretch-activated current in a differentiated mouse epididymal SVF cell at holding potentials of -80 mV and 0 mV (cell-attached configuration). (**d**) Fluo-4-loaded differentiated 3T3-F442A cells transfected with scrambled (control) siRNA or siRNA directed against Piezo1 were exposed to 10 µM Yoda1, and $[Ca^{2+}]_i$ was determined as fluorescence intensity (RFU, relative fluorescence units); shown is a representative experiments of 5 independent experiments. (**e**, **f**) Analysis of adipocyte size after 1, 2, 3, 7 and 14 days of HFD feeding. Shown are the body weight and weight of inguinal and epididymal fad tissue of mice (e) as well as the diameter of adipocytes in sections of the epididymal fat pad in HFD fed mice compared to animals which were maintained on normal chow (f) (n=3 animals (10 sections were analyzed per mouse)). Scale bar in f: 100 µm. (**g**, **h**) Expression of mRNA encoding Piezo1 (g) and protein expression or Piezo1 and GAPDH (h) in vWAT adipocytes and BAT prepared from wild-type (WT) or Ad-Piezo1-KO (KO) mice 14 days after tamoxifen injection (n=3 mice in each group in g; in h, 1 representative of 3 independent experiments is shown). (**i**, **j**) Body weight (i) and weight of inguinal sWAT and epididymal vWAT (j) of wild-type (WT) and Ad-Piezo1-KO (KO) mice under standard diet (SD) (n=8 mice (in h); n=5 mice (in i)). (**k**) Glucose tolerance in wild-type (WT) and Ad-Piezo1-KO mice fed a SD (n=8 mice (WT), n=6 mice (KO)). (**l**) Histological section of epididymal vWAT (left panel) and distribution of size of epididymal vWAT adipocytes (right panel) prepared from wild-type (WT) and Ad-Piezo1-KO mice fed a SD (n=3 mice (WT and KO each)); bar length: 50 µm. Shown are mean values ± s.e.m.; *, $P \leq 0.05$; **, $P \leq 0.01$; ***, $P \leq 0.001$ (two-tailed non-parametric Mann-Whitney U-test). Source data are provided as a Source Data file.

Suppl. Figure 2

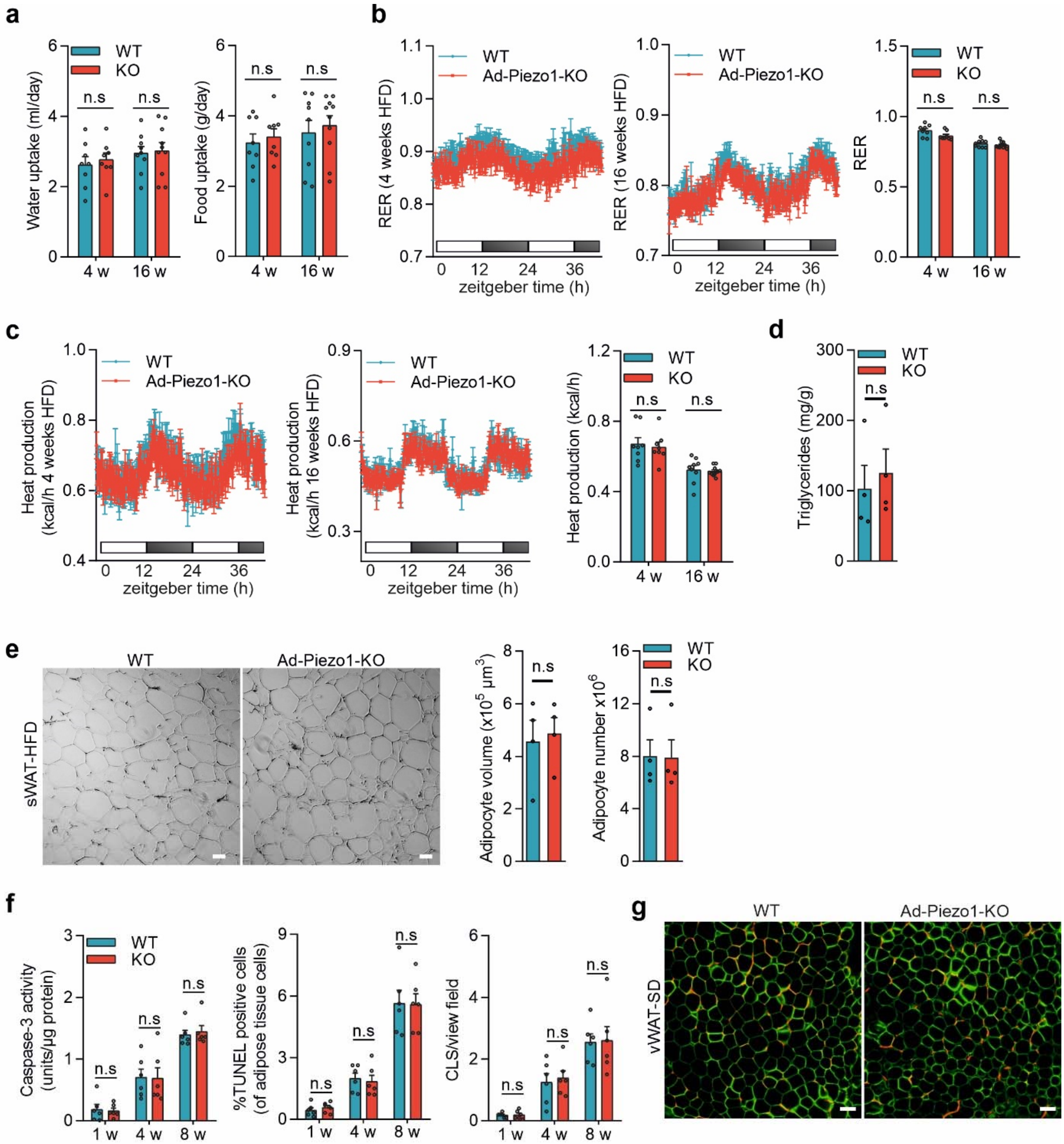

Wang et al., Suppl. Fig. 2

Suppl. Fig. 2. Phenotype of wild-type and Ad-Piezo1-KO mice under HFD and SD. (**a-d**) Water and food uptake (a), respiratory quotient (RER) (b), heat production (c) and liver triglyceride content (d) in wild-type (WT) and Ad-Piezo1-KO mice after 4 and 16 weeks of HFD (n=8 mice (WT and KO 4 weeks of HFD), n=9 mice (WT 16 weeks of HFD), n=10 mice (KO 16 weeks of HFD)). (**e**) H&E-stained inguinal sWAT sections and average volume and total number of adipocytes in inguinal sWAT from wild-type (WT) and Ad-Piezo1-KO (KO) mice after 16 weeks of HFD (n=4 mice (WT and KO each)); bar length: 50 μm. (**f**) Caspase-3 activity, TUNEL staining and crown-like structures (CLS) in vWAT from wild-type (WT) and Ad-Piezo1-KO mice (KO) fed HFD for 1, 4 and 8 weeks (n=6 mice (at least 12 sections per mouse analyzed)). (**g**) Representative images (of 2 independent experiments) of adipocyte

tracing in Adipoq-CreERT2;mT/mG;Piezo1$^{flox/flox}$ (KO) and Adipoq-CreERT2;mT/mG;Piezo1$^{+/+}$ mice (WT) after tamoxifen treatment and 8 weeks of SD in vWAT; bar length: 50 μm. Shown are mean values ± s.e.m; n.s., not significant (two-tailed non-parametric Mann-Whitney U-test). Source data are provided as a Source Data file.#

Suppl. Figure 3

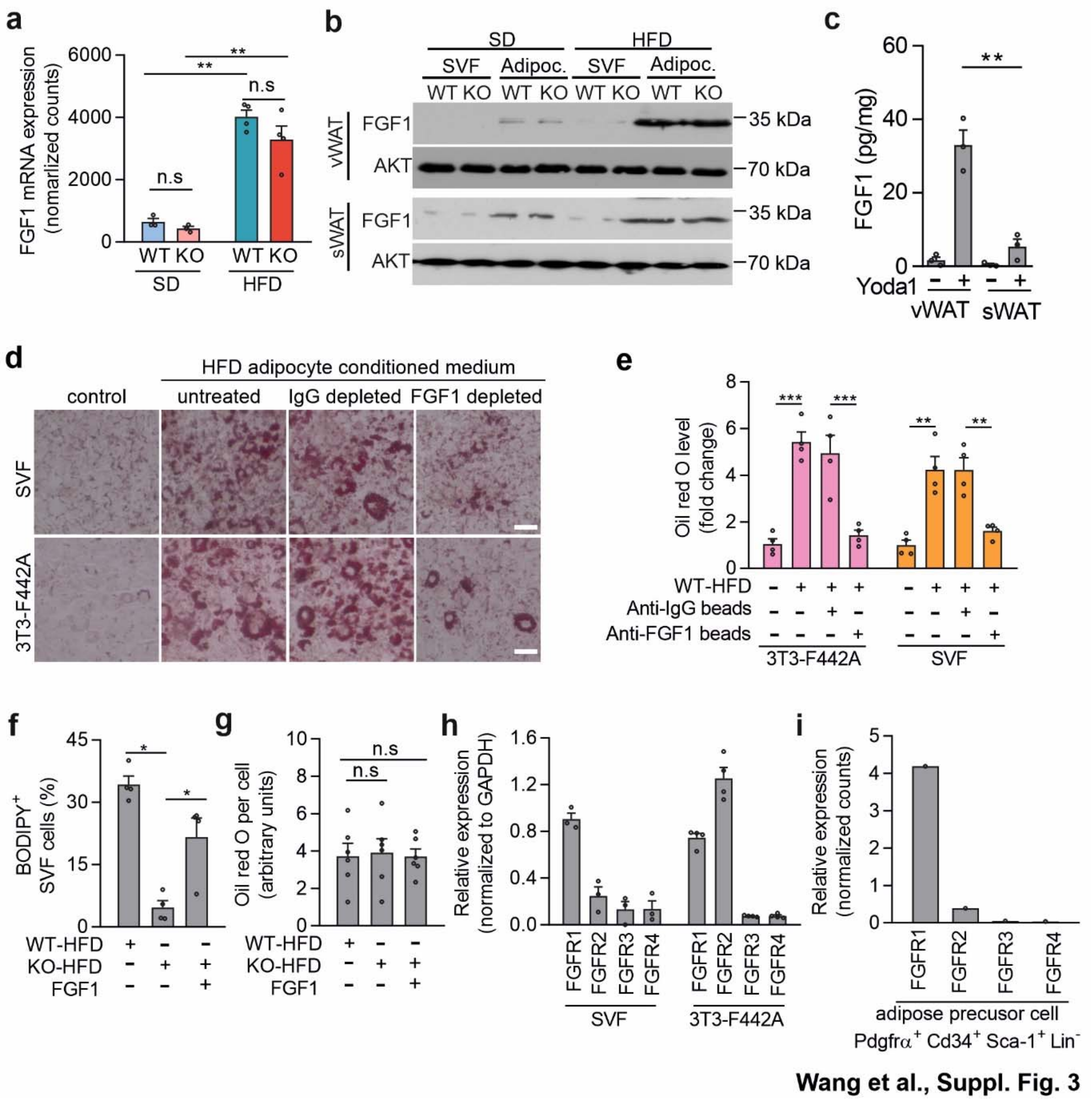

Suppl. Fig. 3. Analysis of preadipocyte proliferation and differentiation in vitro and expression of FGF1 in adipocytes and of FGF receptors in preadipocytes. (**a**) FGF1 expression in vWAT adipocytes prepared from wild-type (WT) and Ad-Piezo1-KO mice (KO) fed SD or HFD for 16 weeks (n=3 mice (SD); n=4 mice (HFD)) analyzed by quantitative RT-PCR. (**b**) Expression of FGF1 and AKT in the SVF or in adipocytes (Adipoc.) of epididymal

WAT (vWAT) or inguinal WAT (sWAT) prepared from animals fed normal chow (standard diet; SD) or HFD for 8 weeks was analyzed by immunoblotting. Shown is a representative of 3 independent experiments. (**c**) Effect of Yoda1 (10 μM) on the release of FGF1 from adipocytes prepared from the vWAT or the sWAT (n=3 mice per group). (**d**,**e**) Effect of pretreatment of conditioned medium of vWAT adipocytes prepared from wild-type mice fed a HFD for 16 weeks with control IgG and anti-FGF1 antibody to deplete FGF1 on its ability to induce differentiation of SVF cells or 3T3-F442A cells. Control: non-conditioned medium. Cells were stained with Oil-red-O (d; shown is a representative of 3 independent experiments). The bar diagram (e) shows the statistical evaluation (n=4). (**f**,**g**) wild-type SVF cells were incubated with conditioned medium (CM) of adipocytes prepared from wild-type (WT) and Ad-Piezo1-KO mice fed HFD for 16 weeks without or with FGF1 (10 nM). Thereafter, cells were collected and one half was analyzed by flow cytometry after staining with BODIPY to determine the number of lipid-positive cells (f). The other half was stained with Oil-red-O to measure the total lipid content, and the average lipid content per cell was then calculated (n=5 mice per group in f; n=4 mice in each group in g). (**h**,**i**) Expression of FGF receptors in the SVF from murine vWAT and undifferentiated 3T3-F442A cells (h) and in Pdgfrα^{+};Cd34^{+};Sca1^{+};Lin^{-} murine preadipocytes (i; data from Tabula Muris Consortium (n = 1)) (n=3 mice (SVF in h); n=4 mice (3T3-F442A in h)). Shown are mean values ± s.e.m.; *, P ≤ 0.05; **, P ≤ 0.01; ***, P ≤ 0.001; n.s., not significant (two-tailed non-parametric Mann-Whitney U-test). Source data are provided as a Source Data file.

Suppl. Figure 4

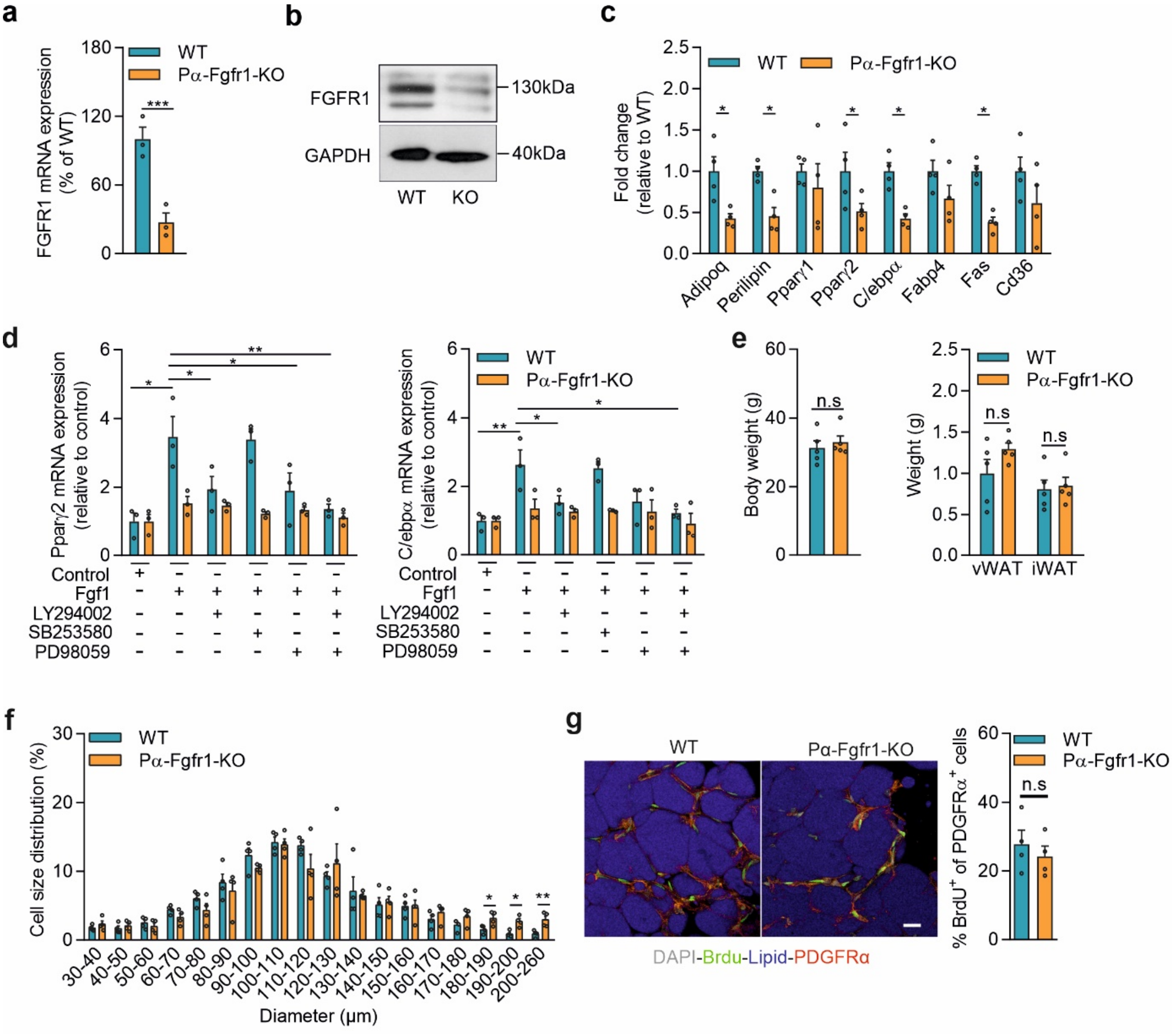

Wang et al., Suppl. Fig. 4

Suppl. Fig. 4. Generation and analysis of mice with preadipocyte-specific Fgfr1 deficiency (Pdgfrα-CreERT2;Fgfr1$^{flox/flox}$). (**a**,**b**) Induction of Pdgfrα-CreER(T2);Fgfr1$^{flox/flox}$ mice (Pα-Fgfr1-KO) with tamoxifen resulted in an about 75 % reduction in FGFR1 expression in Pdgfrα^{+} cells of the SVF (a) and strongly reduced expression of FGFR1 protein levels in Pdgfrα^{+} SVF cells from induced Pα-Fgfr1-KO mice (KO) compared to wild-type animals (WT) as analyzed by immunoblotting (b) (n=3 mice in each group in a; in b, a representative of 2 independent experiments is shown). (**c**, **d**) Effects of conditioned medium from vWAT adipocytes prepared from wild-type mice fed a HFD for 16 weeks (c) or of 10 nM FGF1 (d) on expression of different adipocyte marker genes in cells of the SVF prepared from wild-type mice or from induced Pdgfrα-CreER(T2);Fgfr1$^{flox/flox}$ animals (Pα-Fgfr1-KO) (n=4 mice (WT and KO). In d, the SVF was preincubated with 10 μM LY294002, SB253580 or PD98059 as indicated. (**e**) Body weight and weight of epididymal (vWAT) and inguinal WAT (sWAT) of wild-type (WT) and induced Pdgfrα-CreER(T2);Fgfr1$^{flox/flox}$ animals (Pα-Fgfr1-KO) after 12 weeks of HFD feeding and (n=5 mice (WT and KO)). (**f**) Distribution of size of vWAT

adipocytes prepared from wild-type (WT) and Pα-Fgfr1-KO mice fed HFD for 12 weeks (n=4 mice (WT and KO each)). (**g**) Wild-type (WT) and Pα-Fgfr1-KO mice received BrdU for one week while feeding HFD. Thereafter, animals were killed and vWAT was analyzed. Shown are representative images and quantifications of immunofluorescence staining for BrdU in nuclei of SVF cells stained with an anti-PDGFRα antibody and LipidTOX (lipid); bar length: 50 μm (n=4 mice (at least 15 sections per mouse). Shown are mean values ± s.e.m.; *, $P \leq 0.05$; **, $P \leq 0.01$; ***, $P \leq 0.001$; n.s., not significant (two-tailed non-parametric Mann-Whitney U-test). Source data are provided as a Source Data file.

Suppl. Figure 5

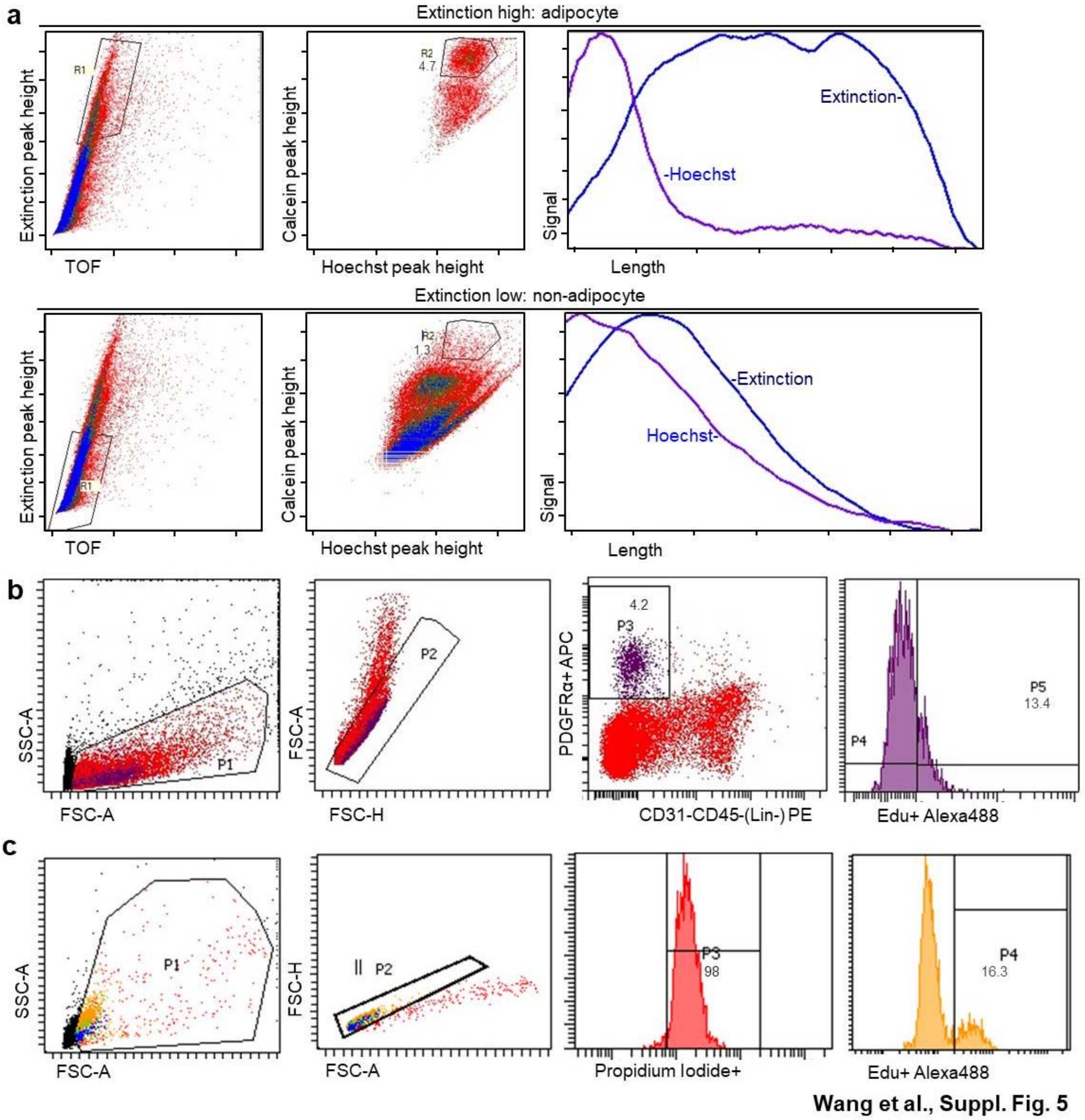

Suppl. Fig. 5. Gating strategies used for flow cytometry. (**a**) Selecting population of cells based on size and extinction and representative profile of single cell in selected gates using a Biosorter. Calcein-AM or GFP fluorescence was collected using BP510/23 nm filter (PMT2) and Hoechst fluorescence using BP 440/30 nm (PMT1). FlowPilot™ software was used to gate the population of adipocytes and single cell profile. Live adipocytes were selected based on Calcein (515 nm) stain and presence of the nucleus (Hoechst, 440 nm). Relative size was determined by the time of flight (TOF) measurement. The optical density of the object was determined by the extinction measurement. (**b**) FSC and SSC gating was performed in order to specifically select adipocyte precursor cells. Edu positive adipocyte precusor cells were gated as: Lin-($CD31^-$;$CD45^-$);$PDGFR\alpha^+$;Edu^+ cells. (**c**) Gating strategy to determine the percentage of Edu^+ adipocyte nuclei that were propidium iodide positive.

Suppl. Table 1

Release of adipogenic mediators and various lipids from adipocytes prepared from wild-type and Ad-Piezo1-KO mice fed a HFD

	WT	Ad-Piezo1-KO		Yoda1 induced release (fold change)
Adiponectin	7,78 ± 2,49	7,48 ± 1,03	ng/ml	1,41 ± 0,51
BMP2	0,14 ± 0,04	0,17 ± 0,04	ng/ml	1,02 ± 0,42
BMP3	25,82 ± 5,35	20,12 ± 4,23	pg/ml	1,31 ± 0,78
IGF-1	6,26 ± 0,32	4,42 ± 0,43	ng/ml	1,01 ± 0,16
FGF-1	27,7 ± 5,67	4,33 ± 2,41*	pg/ml	8,81 ± 2,35**
FGF-2	10,8 ± 1,23	10,2 ± 1,56	pg/ml	1,12 ± 0,11
Jag-1	106,3 ± 7,9	91,2 ± 9,8	pg/ml	1,01 ± 0,16
TGF-β	8,6 ± 0,48	8,5 ± 0,36	ng/ml	1,12 ± 0,23
WNT-5a	9,8 ± 0,29	12,81 ± 0,82	pg/ml	0,96 ± 0,27
WNT-10b	0,04 ± 0,005	0,05 ± 0,006	pg/ml	1,05 ± 0,13
TXB2	152,16 ± 11,09	183,45 ± 5,7	ng/ml	0,71 ± 0,34
PGF2α	281,82 ± 14,47	192,49 ± 32,1	ng/ml	0,51 ± 0,14
PGH2	0,27 ± 0,07	0,29 ± 0,06	ng/ml	0,97 ± 0,22
PGE1	15,89 ± 6,5	20,34 ± 10,32	ng/ml	1,26 ± 0,65
PGE2	20,86 ± 5,33	83,28 ± 12,51*	ng/ml	1,08 ± 0,45
PGD2	23,38 ± 2,63	25,85 ± 1,82	ng/ml	0,89 ± 0,19
PGJ2	38,27 ± 4,39	44,87 ± 6,91	ng/ml	N/A
PGA2	17,94 ± 5,4	23,15 ± 4,96	ng/ml	N/A
LTB4	5,74 ± 2,74	3,66 ± 1	ng/ml	0,98 ± 0,64
LXA4	32.36 ± 31.39	26.23 ± 13.61	ng/ml	N/A
12,13-diHOME	75,29 ± 49,2	48,5 ± 29,4	ng/ml	1,12 ± 0,31
12-HHTrE	0,02 ± 0,01	0,01 ± 0,008	ng/ml	0,94 ± 0,45
13S-HOTrE	29,4 ± 9,75	30,1 ± 7,15	ng/ml	0,93 ± 0,77
9,10-diHOME	46,1± 23,2	45,4 ± 25,4	ng/ml	1,11 ± 0,35
11,12-diHETrE	44,7 ± 16,4	44,9 ± 15,2	ng/ml	0,74 ± 0,17
14,15-diHETrE	21,6 ± 13,9	24,5 ± 13,4	ng/ml	0,98 ± 0,63
14,15-diHETE	62,8 ± 30,2	46,8 ± 37,5	ng/ml	0,94 ± 0,3
17,18-diHETE	22,2 ± 17,8	18,3 ± 13,3	ng/ml	0,86 ± 0,49
19,20-DiHDPA	53,9 ± 15,7	53,6 ± 13,7	ng/ml	0,96 ± 0,13
19-HETE	121,7 ± 13,8	152,1 ± 34,1	ng/ml	1,22 ± 0,23
13-HODE	29,4 ± 9,8	30,1 ± 7,2	ng/ml	0,92 ± 0,38
9-HODE	40,1 ± 44,8	47,1 ± 22,2	ng/ml	1,03 ± 0,56
15S-HETE	2,41 ± 1,73	11,4 ± 24,3	ng/ml	0,88 ± 0,51
17-HDHA	0,011 ± 0,004	0,013 ± 0,005	ng/ml	1,16 ± 0,46
13-HpODE	0,005 ± 0,006	0,003 ± 0,003	ng/ml	1,01 ± 0,54
9-HpODE	0,037 ± 0,029	0,018 ± 0,009	ng/ml	1,09 ± 0,31
15-HETrE	0,049 ± 0,011	0,091 ± 0,109	ng/ml	1,23 ± 0,42
12-HETE	0,42 ± 0,19	0,92 ± 0,34	ng/ml	7,48 ± 1,40
8-HETE	3,7 ± 1,4	3,8 ± 2,6	ng/ml	0,92 ± 0,62
5-HETE	29,8 ± 21,7	25,8 ± 7,3	ng/ml	1,25 ± 0,79
19,20-EpDPE	0,023 ± 0,01	0,024 ±0,01	ng/ml	0,87 ± 0,28
14-HDoHE	0,056 ± 0,052	0,047 ± 0,017	ng/ml	1,13 ± 0,46
8-HETrE	0,014 ± 0,007	0,015 ± 0,008	ng/ml	N/A

9,10-EpOME	0,67 ± 0,44	1,01 ± 0,34	ng/ml	0,95 ± 0,44
12,13-EpOME	0,17 ± 0,07	0,25 ± 0,06	ng/ml	0,96 ± 0,45
16,17-EpDPE	4,13 ± 0,01	6,69 ± 1,44	ng/ml	1,00 ± 0,59
11,12-EET	0,95 ± 1,0	2,11 ± 2,9	ng/ml	0,86 ± 0,59
14,15-EET	0,05 ± 0,02	0,02 ± 0,004	ng/ml	0,96 ± 0,54
5,6-EET	0,58 ± 0,34	1,53 ± 0,58	ng/ml	0,93 ± 0,56
EPA	255,7 ± 26,2	383,7 ± 22,6	ng/ml	1,02 ± 0,21
Linoleic acid	824,5 ± 58,7	675,2 ± 45,4	ng/ml	0,95 ± 0,26
Arachidonic acid	422,5 ± 31,1	503,9 ± 29,3	ng/ml	0,79 ± 0,39
DHA	103,9 ± 72,6	128,3 ± 58,3	ng/ml	0,91 ± 0,51
DHGLA	13,6 ± 9,2	17,3 ± 10,9	ng/ml	# 0,76 ± 0,34

Shown are mean values ± s.e.m.; *, P ≤ 0.05; **, P ≤ 0.01 (two-tailed non-parametric Mann-Whitney U-test)

#
