# Supplementary material for: Adipocyte Piezo1 mediates obesogenic adipogenesis through the FGF1/FGFR1 signaling pathway in mice
Source: Nat Commun. 2020 May 8;11:2303. doi: 10.1038/s41467-020-16026-w (PMC7211025; doi:10.1038/s41467-020-16026-w)
Supplement: Supplementary file 2 — Reporting Summary [file 41467_2020_16026_MOESM2_ESM.pdf]

nature research

Corresponding author(s): Stefan Offermanns, ShengPeng Wang

Last updated by author(s): 15/03/20

Reporting Summary

Nature Research wishes to improve the reproducibility of the work that we publish. This form provides structure for consistency and transparency in reporting. For further information on Nature Research policies, see Authors & Referees and the Editorial Policy Checklist.

Statistics

For all statistical analyses, confirm that the following items are present in the figure legend, table legend, main text, or Methods section.

n/a	Confirmed	
☐	☒	The exact sample size (n) for each experimental group/condition, given as a discrete number and unit of measurement
☐	☒	A statement on whether measurements were taken from distinct samples or whether the same sample was measured repeatedly
☐	☒	The statistical test(s) used AND whether they are one- or two-sided<br>*Only common tests should be described solely by name; describe more complex techniques in the Methods section.*
☒	☐	A description of all covariates tested
☐	☒	A description of any assumptions or corrections, such as tests of normality and adjustment for multiple comparisons
☐	☒	A full description of the statistical parameters including central tendency (e.g. means) or other basic estimates (e.g. regression coefficient) AND variation (e.g. standard deviation) or associated estimates of uncertainty (e.g. confidence intervals)
☐	☒	For null hypothesis testing, the test statistic (e.g. F, t, r) with confidence intervals, effect sizes, degrees of freedom and P value noted<br>*Give P values as exact values whenever suitable.*
☒	☐	For Bayesian analysis, information on the choice of priors and Markov chain Monte Carlo settings
☒	☐	For hierarchical and complex designs, identification of the appropriate level for tests and full reporting of outcomes
☒	☐	Estimates of effect sizes (e.g. Cohen's d, Pearson's r), indicating how they were calculated

Our web collection on statistics for biologists contains articles on many of the points above.

Software and code

Policy information about availability of computer code

Data collection	qRT-PCR: LightCycler 480 software 1.5.0SP3<br>imaging, histology analysis: Leica LAS-AF, Leica-SP8 FLIM, Olympus cell^R software 3.2, Keyence BZ-9000 software.<br>spectrophotometry: SkanIt for Multiskan GO 2.0., SoftMax® Pro Software, Analyst 1.6.2 and MultiQuant 3.0<br>FACS data acquisition: Becton Dickinson FACSDiva, Union Biometrica Profiler II™<br>Calorimetry: PhenoMaster-Software (TSE-Systems)<br>MILLIPLEX Analyst 5.1 software (Merck Millipore)
Data analysis	statistics and graphs: Prism5 or Prism6 (GraphPad) , Microsoft office Excel 2016<br>image analysis: ImageJ/FIJI

For manuscripts utilizing custom algorithms or software that are central to the research but not yet described in published literature, software must be made available to editors/reviewers. We strongly encourage code deposition in a community repository (e.g. GitHub). See the Nature Research guidelines for submitting code & software for further information.

Data

Policy information about availability of data

All manuscripts must include a data availability statement. This statement should provide the following information, where applicable:

- Accession codes, unique identifiers, or web links for publicly available datasets
- A list of figures that have associated raw data
- A description of any restrictions on data availability

The source data underlying Figs. 1b,d,e,f, 2a,b,d-l, 3a-c and e, 4b,c,e-h, 5a-c, 6a-g and i-k and Supplementary Figs. 1a,e-l, 2a-f, 3a,b,d-h, 4a-g are provided as Source Data file.

Field-specific reporting

Please select the one below that is the best fit for your research. If you are not sure, read the appropriate sections before making your selection.

☒ Life sciences ☐ Behavioural & social sciences ☐ Ecological, evolutionary & environmental sciences

For a reference copy of the document with all sections, see nature.com/documents/nr-reporting-summary-flat.pdf

Life sciences study design

All studies must disclose on these points even when the disclosure is negative.

Sample size	Sample size was determined on basis of trial experiments or experiments done previously (see under "Statistical Analysis" in Methods section).
Data exclusions	Samples were excluded in cases where cDNA quality or tissue quality after processing was poor (below commonly accepted standards). Animals were excluded from experiments if they showed any signs of sickness (weight loss more than 20 %, skin infection, shaggy fur, apathy, loss of / or reduced movements, any motoric abnormality, atypical body posture, abnormal breathing, unusual aggressiveness during handling) (see Methods under "Statistical Analysis").
Replication	Each experiment was repeated at least twice under independent conditions.
Randomization	No randomization was used for samples. In animal experiments, mice were caged with blinded cage numbers and random orders (see Methods under "Statistical Analysis").
Blinding	The investigator was blinded to the group allocation and during the experiment (see Methods under "Statistical Analysis"). In animal experiments, mice were caged with blinded cage numbers and random orders (see Methods under "Statistical Analysis").

Reporting for specific materials, systems and methods

We require information from authors about some types of materials, experimental systems and methods used in many studies. Here, indicate whether each material, system or method listed is relevant to your study. If you are not sure if a list item applies to your research, read the appropriate section before selecting a response.

Materials & experimental systems

n/a	Involved in the study	
☐	☒	Antibodies
☐	☒	Eukaryotic cell lines
☒	☐	Palaeontology
☐	☒	Animals and other organisms
☐	☒	Human research participants
☒	☐	Clinical data

Methods

n/a	Involved in the study	
☒	☐	ChIP-seq
☐	☒	Flow cytometry
☒	☐	MRI-based neuroimaging

Antibodies

Antibodies used	All antibodies used in this study are listed in the the Methods section with catalog number and/or clone number. BODIPY (Invitrogen, catalog number D3922), DAPI (Invitrogen, catalog number D1306) and anti-Ki67 antibodies (Abcam, catalog number ab15580),Anti-Piezo1 (Proteintech catalog number 15939-1-AP), anti-GAPDH (Cell Signaling Technology#2188), anti-FGFR1(Cell Signaling Technology #9740) and anti-AKT antibodies(Cell Signaling Technology #9272),anti-FGF1(Santa Cruz Biotechnology #sc-55520),anti-Perilipin-1 (Abcam # ab61682), anti-BrdU (Abcam # ab6326), anti-PDGFRα (R&D #AF1062), anti-PDGFRα (APC, eBioscience #17-1401), anti-CD34 (AF700, eBioscience #57-0341), anti-CD31 (efluor450, eBioscience #48-0311-80) and anti-CD45 (efluor450, eBioscience #48-0451-80), mouse IgG2b (Santa Cruz Biotechnology #sc-3879), LipidTOX™ (Molecular Probes #H34475),CellMask Orange (Molecular Probes #C10045),goat anti-mouse AF-546, (Molecular Probes # A11003), donkey anti-rabbit AF-488 (Molecular Probes #A11008),rabbit anti-goat AF-594 (Molecular Probes #A11080),Hoechst 34580 (Invitrogen #H21486) and Calcien-AM (BD #564061).
Validation	All antibodies have been validated according to instruction on the manufacturers´ website and in eukaryotic cell lines after gene silencing. Absence of specific band was considered a consequence of gene silencing and validation of the specificity of the antibody (gene silencing efficiency was verified by qRT-PCR).

Eukaryotic cell lines

Policy information about cell lines

Cell line source(s)	3T3-F442A cell line was obtained from European Collection of Authenticated Cell Cultures (ECACC)

Authentication	Cell lines were not authenthicated
Mycoplasma contamination	Mycoplasma test was performed twice each year.Cell lines used in this study tested negative for mycoplasma.
Commonly misidentified lines (See ICLAC register)	No commonly misidentified cell lines were used

Animals and other organisms

Policy information about studies involving animals; ARRIVE guidelines recommended for reporting animal research

Laboratory animals	Stated in Materials and Methods, C57BL/6N male and female animals (8–24 weeks old) were used. Mice were housed under a 12-h light–dark cycle with free access to food and water and under specific pathogen–free conditions if not stated otherwise. Mice carrying a floxed allele of the gene encoding Piezo1 and Fgfr1 as well as the Cre reporter line Gt(ROSA)26Sortm4(ACTB-tdTomato,-EGFP)Luo/J (mT/mG) were obtained from The Jackson Laboratory. Animals expressing the lacZ gene under the control of the Piezo1 promoter were obtained from the Knockout Mouse Project (KOMP). Mice which allow for tamoxifen-dependent adipocyte-specific Cre activation have been described before and Pdgfrα-CreERT2 animals were provided by the RIKEN BRC through the National BioResource Project of the MEXT/AMED, Japan (RBRC09616).
Wild animals	This study did not involve wild animals
Field-collected samples	The study did not involve samples collected from the field
Ethics oversight	All procedures of animal care and use in this study were approved by the local animal ethics committees (Regierungspräsidium Darmstadt, Germany and Ethical Committee of Xi'an Jiaotong University).

Note that full information on the approval of the study protocol must also be provided in the manuscript.

Human research participants

Policy information about studies involving human research participants

Population characteristics	Nr1:Female, 42 yo, Chinese, BMI 32.7<br>Nr2:Female, 54 yo, Chinese, BMI 29.9<br>Nr3:Female, 23 yo, Chinese, BMI 29.3<br>Nr4:Male, 45 yo, Chinese, BMI 22.5<br>Nr5:Male, 55 yo, Chinese, BMI 21.5
Recruitment	Written informed consent was obtained from all subjects before their participation. The visceral adipose tissues were donated from either obese patients (BMI≥28) who underwent laparotomy surgery or normal control subjects who underwent non-laparotomy surgeries. Patients were anonymous to the researchers except for information of gender, age, and BMI.
Ethics oversight	The work on human adipocyte samples was approved by the ethical committee of Xi'an Jiaotong University (XJTU2018-249 and XJTU2019-12) and conforms to the guidelines of the 2000 Helsinki declaration. Written informed consent was obtained from all subjects before their participation.

Note that full information on the approval of the study protocol must also be provided in the manuscript.

Flow Cytometry

Plots

Confirm that:

- ☒ The axis labels state the marker and fluorochrome used (e.g. CD4-FITC).
- ☒ The axis scales are clearly visible. Include numbers along axes only for bottom left plot of group (a 'group' is an analysis of identical markers).
- ☒ All plots are contour plots with outliers or pseudocolor plots.
- ☒ A numerical value for number of cells or percentage (with statistics) is provided.

Methodology

Sample preparation	adipocyte and SVF preparation were descripted listed in the Methods section
Instrument	BD FACS Canto II , BioSorter large particle flow cytometer (Union Biometrica)
Software	Becton Dickinson FACSDiva, FlowPilot™ software
Cell population abundance	Ex vivo EdU experiments: No cell sorting was performed.

Gating strategy

Ex vivo EdU experiments: FSC and SSC gating was performed in order to specifically select adipocyte precusor cells, according to publication (Church et al. Methods Enzymol. 2014 ; 537: 31–46. doi:10.1016/B978-0-12-411619-1.00003-3.) as the gating strategy for adipocyte presucor (highly enriched Cd31-;Cd45-;PDGFRα+). Boundaries between positive and negative were defined according to the unstained sample, single stainings and fluorescence minus one controls in order to prevent the selection of unspecific signal or autofluorescence.
Adipocyte size experiments: Using a large partical sorter live adipocytes were isolated based on Calcein membrane and Hoechst nuclear staining as reported in Methods section. Size and optical density were used to exclude debris and aggregates.

☒ Tick this box to confirm that a figure exemplifying the gating strategy is provided in the Supplementary Information.
